# Orientation selective DBS of entorhinal cortex and medial septal nucleus modulates activity of rat brain areas involved in memory and cognition

**DOI:** 10.1038/s41598-022-12383-2

**Published:** 2022-05-20

**Authors:** Lin Wu, Antonietta Canna, Omar Narvaez, Jun Ma, Sheng Sang, Lauri J. Lehto, Alejandra Sierra, Heikki Tanila, Yuan Zhang, Olli Gröhn, Walter C. Low, Pavel Filip, Silvia Mangia, Shalom Michaeli

**Affiliations:** 1 Center for Magnetic Resonance Research, University of Minnesota, Minneapolis, MN USA; 2 University of Campania “Luigi Vanvitelli”, Naples, Italy; 3 A. I. Virtanen Institute for Molecular Sciences, University of Eastern Finland, Kuopio, Finland; 4 Department of Neurosurgery, University of Minnesota, Minneapolis, USA; 5 Division of Biostatistics, School of Public Health, University of Minnesota, Minneapolis, MN USA; 6 Department of Neurology, First Faculty of Medicine and General University Hospital, Charles University, Prague, Czech Republic; 7 Radiology Department, Center for MR Research, University of Minnesota, 2021 6th St. SE, Minneapolis, MN 55455 USA

**Keywords:** Neural circuits, Biomedical engineering

## Abstract

The recently introduced orientation selective deep brain stimulation (OS-DBS) technique freely controls the direction of the electric field’s spatial gradient by using multiple contacts with independent current sources within a multielectrode array. The goal of OS-DBS is to align the electrical field along the axonal track of interest passing through the stimulation site. Here we utilized OS-DBS with a planar 3-channel electrode for stimulating the rat entorhinal cortex (EC) and medial septal nucleus (MSN), two promising areas for DBS treatment of Alzheimer’s disease. The brain responses to OS-DBS were monitored by whole brain functional magnetic resonance imaging (fMRI) at 9.4 T with Multi-Band Sweep Imaging with Fourier Transformation (MB-SWIFT). Varying the in-plane OS-DBS stimulation angle in the EC resulted in activity modulation of multiple downstream brain areas involved in memory and cognition. Contrary to that, no angle dependence of brain activations was observed when stimulating the MSN, consistent with predictions based on the electrode configuration and on the main axonal directions of the targets derived from diffusion MRI tractography and histology. We conclude that tuning the OS-DBS stimulation angle modulates the activation of brain areas relevant to Alzheimer’s disease, thus holding great promise in the DBS treatment of the disease.

## Introduction

Although Deep Brain Stimulation (DBS) is an established therapeutic modality for the treatment of movement disorders, its utility for Alzheimer’s disease is also becoming increasingly evident^1–3^. The entorhinal cortex (EC)^4^, fornix^5^, nucleus basalis of Meynert (NBM)^6^, anterior nucleus of the thalamus^7^ and medial septal nucleus (MSN)^8^ are promising DBS targets for improving dysfunctional memory and other cognitive functions. Putative mechanisms of actions include alteration of neuronal firing patterns, increase communication across several brain regions, amplification of synaptic plasticity^9^ and induction of neurogenesis^7^. In particular, DBS of the EC^10^ and MSN^8^ induces neurogenesis by promoting neural stem proliferation in the dentate gyrus subgranular zone of the hippocampus (HC). After DBS of the EC in mice, the newly born nerve cells can integrate into the neural circuitry of the dentate gyrus, survive long term, and there is a relationship between stimulation-induced promotion of neurogenesis, differentiation of neural stem cells to mature dentate granule cells, and enhanced spatial memory^10^. Experimental work in rodent models further indicated that fornix DBS may lead to improved memory via elevation of acetylcholine release in HC^11^ and induction of growth factors^12^.

Several functional features of the EC and MSN corroborate their use as potential targets for the treatment of Alzheimer’s disease. In particular, the EC provides the major cortical input to the HC, and transgenic mice in which these inputs are inhibited display impaired temporal association memory^13^. On the other hand, MSN acts as a “pacemaker” in regulating hippocampal theta oscillations^14–16^ and its stimulation prior to a spatial working memory task has been shown to enhance hippocampal theta activity and improve spatial working memory^17^. In brain slices, a cholinergic agonist amplifies the theta rhythm and increases the sensitivity of HC synapses to undergo long-term potentiation or depression^18^. Thus, MSN stimulation may improve memory encoding by strengthening the theta rhythm through augmenting the cholinergic input to the HC. Moreover, in Alzheimer’s disease mouse model, optogenetic stimulation of medial septal parvalbumin neurons could restore the hippocampal gamma oscillations despite significant plaque deposition^19^. Beyond Alzheimer’s disease, MSN stimulation can be a valuable “proxy intervention”^20^ also for epilepsy, since structures such as the hippocampus and entorhinal cortex are often involved in the disease. Both optogenetic and electrical stimulation of the MSN have indeed been shown to reduce seizures in HC and rescue memory function in epileptic rats^20–22^. DBS of the MSN thus yields great promise for translation in patients with temporal lobe epilepsy who have memory deficits.

Initial clinical trials have been conducted to assess the efficacy of DBS in patients with Alzheimer’s disease^23^. In a pilot study of six patients with mild to moderate Alzheimer's disease, improved cognitive performance and enhanced ^18^F-glucose uptake in the brain were observed following stimulation of the NBM^24^. Other studies have suggested that targeting the fornix leads to improved cognition^25^, enhanced cortical and hippocampal ^18^F-glucose uptake^26^, and increased hippocampal volumes^27^. However, a subsequent multicenter phase II trial on fornix stimulation found no significant differences between the DBS and control groups with respect to primary cognitive outcomes^28,29^*.* This lack of DBS efficacy may be at least partially attributed to inadequate target selectivity during DBS.

To advance the spatial selectivity of neuronal modulation with DBS, our group pioneered a novel orientation-selective strategy for DBS, entitled OS-DBS^30^. This strategy entails that, by using multiple contacts with independent current sources within a multielectrode array, the electric field can be oriented along any desired orientation in space, such that axons parallel to the electric field spatial gradients are preferentially activated. The OS-DBS technique has been successfully used in rodents with a planar three-channel electrode to attain in-plane reorientation of the primary direction of the electrical field in stimulation sites encompassing the corpus callosum^30^, the infralimbic cortex (IL)^31^, and the subthalamic nucleus (STN)^32^. Additionally, we have demonstrated that OS-DBS shows promise to significantly improve the clinical outcomes of DBS therapy when using commercially available DBS leads in clinical settings^33^.

In this study, OS-DBS with a three-channel electrode was utilized for stimulation of the rat EC and MSN with the goal of modulating the activation of brain networks connected to the stimulation sites. The stimulation effects were monitored by means of whole brain fMRI with Multi-Band SWeep Imaging with Fourier Transformation (MB-SWIFT). Such imaging modality operates with virtually no echo time and large bandwidth (BW), and thus minimizes artefacts from implanted electrodes and motion^34,35^. The effect of OS-DBS on the fMRI maps was evaluated in single subjects and at group level. Moreover, a region of interest (ROI) analysis was performed to quantify fMRI activation strength in downstream areas critical to Alzheimer’s disease, and/or connected to the targets. For the EC OS-DBS, the primary ROIs included dorsal and ventral hippocampus (DHC and VHC, respectively), subiculum (Sub), as well as other areas connected to the EC such as perirhinal cortex (PrC), piriform cortex (Pir), amygdala (Amg) and insula (Ins). For the MSN case, the primary ROIs included the DHC and VHC, Sub, and other areas connected to the MSN such as the interpeduncular nucleus (IP), the lateral hypothalamus (LH), the mammillary bodies (MM) and supramamillary nuclei (SuM). Finally, in order to substantiate the fMRI findings during OS-DBS, we characterized the main axonal orientations of the EC and MSN by diffusion MRI tractography and histological assessments.

## Results

**Figure 1:**
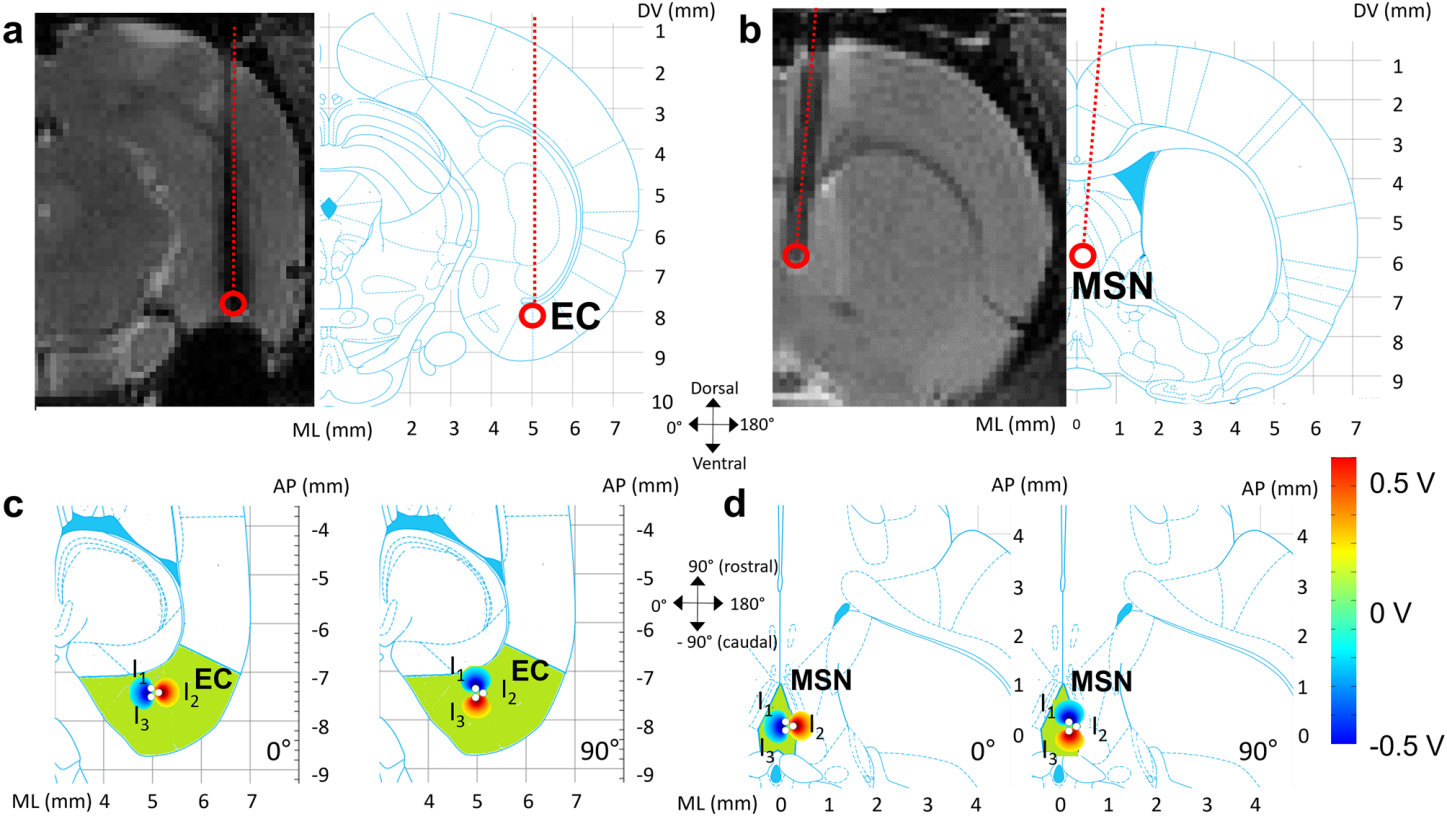
Electrode locations and electrical field distributions. Illustration of the electrode location in the EC (**a**) and MSN (**b**) on a coronal T2-weighted MRI image (left) and on a corresponding section (right) of the rat brain taken with permission from the Rat Brain in Stereotaxic Coordinates (6th Edition) atlas^36^. Schematics of the field distribution around the 3-channel electrode for two representative angles superimposed on an anatomical horizontal section (corresponding to an axial MRI view) of the rat brain taken from the atlas are shown for EC (**c**) and MSN (**d**) stimulation. Field distributions were obtained with COMSOL 5.4 (COMSOL, Stockholm, Sweden). 0°/180° corresponds to the mediolateral direction and 90°/− 90° corresponds to the rostrocaudal direction on the horizontal plane. The level of current and the diameter of the electrode bundle were set to 1 mA and ~ 350 µm, respectively, resembling the values that were used in the experiments. Brain images are displayed in neurological convention (left side of the image corresponds to the left side of the brain).

All fMRI sessions conducted during OS-DBS in both EC and MSN (Fig. 1) were successfully completed and provided data of sufficient quality for subsequent fMRI analyses. However, in two rats out of 12 undergoing OS-DBS in the EC, the electrode was determined to be out of target based on the estimated dorsal–ventral (DV) location of the electrode tip obtained by overlapping the MRI images with the rat brain atlas. Therefore, these rats were excluded from further analyses. In the remaining 10 rats of the EC group, the electrode DV coordinates were 8.1 ± 0.2 mm from dura, and the delivered current amplitudes were 1.7 ± 0.4 mA (Supplementary Fig. 1). For the 8 rats of the MSN group, the DV coordinates were 6.0 ± 0.2 mm, and the current amplitudes 1.0 ± 0.3 mA (Supplementary Fig. 2).

**Figure 2:**
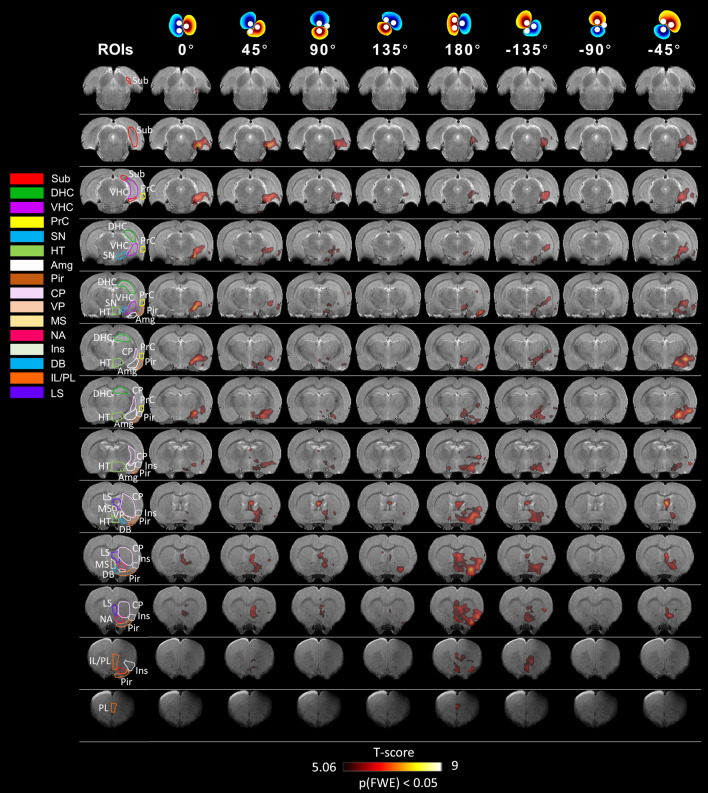
Main effects of OS-DBS in the right EC for all stimulation angles (n = 10). Activation maps were obtained by the one-way within subject ANOVA model (p ≤ 0.05, FWE corrected). Amg: amygdala, CP: caudate-putamen, DB: diagonal band, DHC: dorsal hippocampus, VHC: ventral hippocampus, HT: hypothalamus, IL/PL: infralimbic/prelimbic cortices, Ins: insula, LS: lateral septum, MS: medial septum, NA: nucleus accumbens, Pir: piriform cortex, PrC: perirhinal cortex, SN: substantia nigra, Sub: subiculum, VP: ventral pallidum. Coronal brain images are displayed in neurological convention (left corresponds to the left side of the brain). Stimulation angles are shown on top. Stimulation frequency was 20 Hz.

For the EC stimulation, we first conducted preliminary fMRI studies in one rat (not shown) at various frequencies (namely 20 Hz, 70 Hz, and 130 Hz) and found that robust fMRI response in the hippocampus was detected at 20 Hz, which was thus chosen for the rest of the study. This was also the frequency that most effectively activated HC with perforant path stimulation in an earlier study^37^. OS-DBS of the EC at 20 Hz robustly activated numerous downstream brain regions. The activated areas were mainly located on the right hemisphere in accordance with the stimulation side. Varying the stimulation angle led to brain network modulations which could be appreciated in single subjects (Supplementary Fig. 3), as well as at group level (Fig. 2) despite a substantial inter-subject variability. No group main effects were seen at − 90°, although 4 out of 10 rats exhibited brain activation at this angle. Significant group main effects in VHC, Sub, Amg, and PrC occurred at 0° and − 45°, while group main effects in Pir and Ins generally occurred at 180°. In addition, a significant main effect was found in VHC at − 135°. Beside activation in the primary ROIs, the main effect maps revealed significant activations at various angles also in other brain areas, including caudate-putamen (CP), diagonal band (DB), hypothalamus (HT), infralimbic/prelimbic cortices IL/PL, lateral septum (LS), medial septum (MS), nucleus accumbens (NA), substantia nigra (SN), and ventral pallidum (VP).

**Figure 3:**
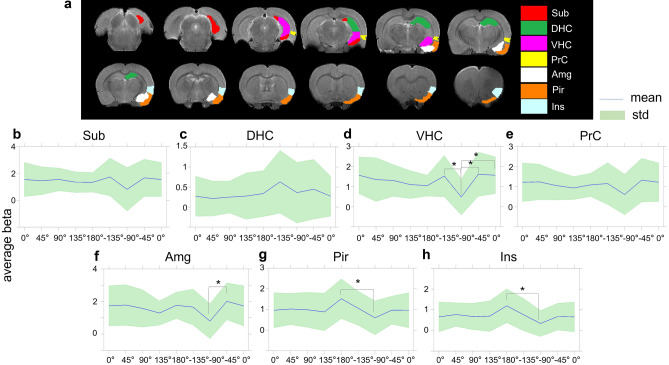
ROI analyses of OS-DBS in the right EC (n = 10). The locations of the primary ROIs are shown on T2-weighted MRI images (**a**). Average beta values are shown for the ROI in the subiculum, Sub (**b**); dorsal hippocampus, DHC (**c**); ventral hippocampus (VHC) **(d)**; perirhinal cortex, PrC (**e**); amygdala, Amg (**f**); piriform cortex, Pir (**g**); and insula, Ins (**h**). The blue line and green area represent the mean value and standard deviation among rats, respectively. *p < 0.05, corrected (linear mixed model comparisons vs −90°, adjusted for Bonferroni multiple comparisons correction). Coronal brain images are displayed in neurological convention (left corresponds to the left side of the brain). Stimulation frequency was 20 Hz.

Average beta-values in the primary ROIs (Fig. 3) generally confirmed the stimulation angle effects seen in the group fMRI maps. The linear mixed model revealed several significant statistical differences after Bonferroni multiple comparison correction. Namely, higher beta-values, indicative of stronger activation, were observed in VHC (Fig. 3d) at − 45° vs − 90° (p = 0.004, corrected), at − 135° vs − 90° (p = 0.01, corrected), and at 0° vs − 90° (p = 0.007, corrected). Higher beta-values were also observed in Amg (Fig. 3f) at − 45° vs − 90° (p = 0.017, corrected), in Pir (Fig. 3g) at 180° vs − 90° (p = 0.017, corrected), and Ins (Fig. 3h) at 180° vs − 90° (p = 0.005, corrected). While modulations were seen also for the DHC, Sub, and PrC, they did not reach statistical significance.

**Figure 4:**
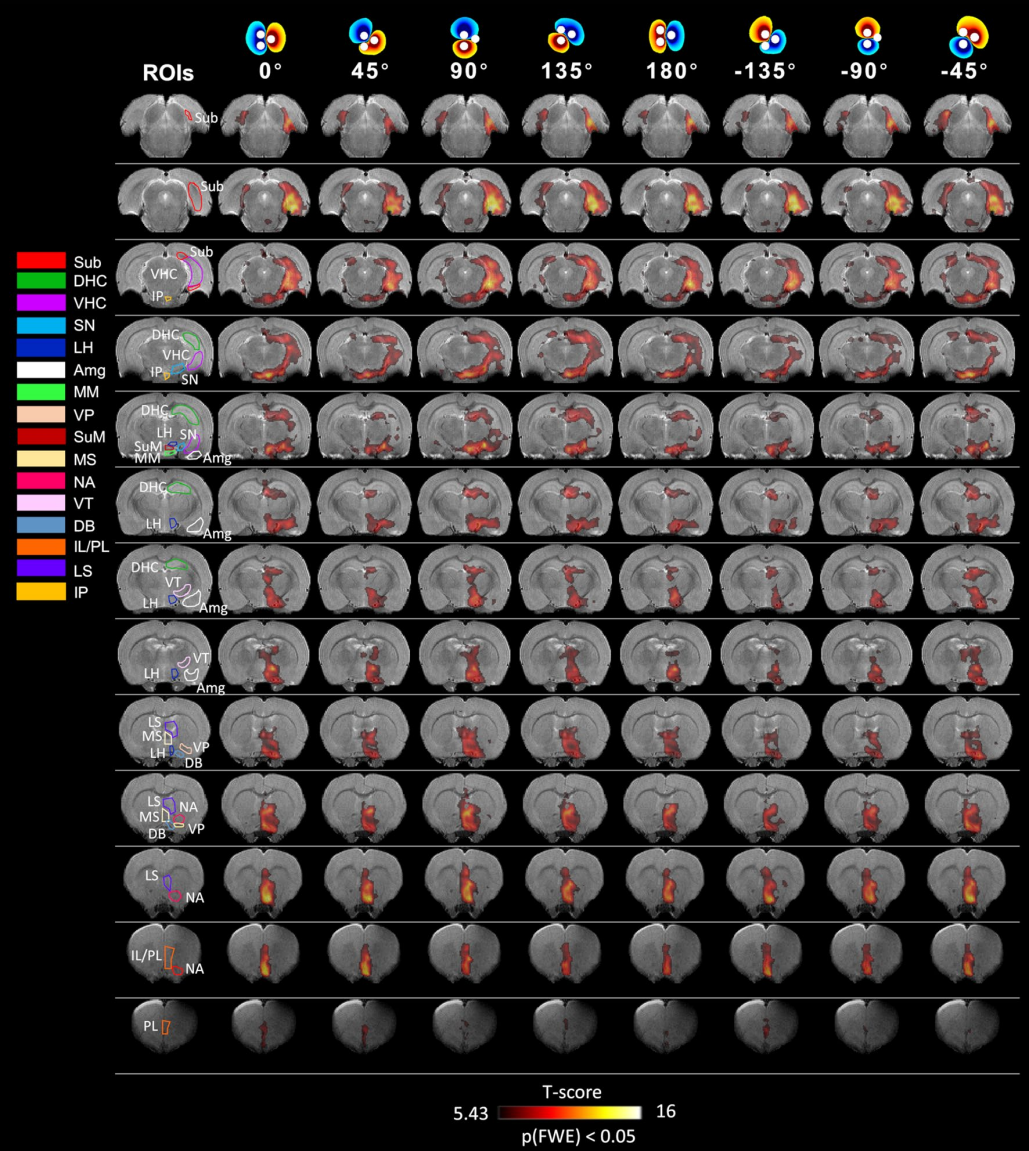
Main effects of OS-DBS in the MSN for all stimulation angles (n = 8). Maps were obtained by the one-way within subject ANOVA model (p < 0.05, FWE corrected). Stimulation angles are indicated on the top. Amg: amygdala, DB: diagonal band, DHC: dorsal hippocampus, VHC: ventral hippocampus, LH: lateral hypothalamus, IL/PL: infralimbic/prelimbic cortices, LS: lateral septum, MM: mammillary bodies; MS: medial septum, NA: nucleus accumbens, SN: substantia nigra, Sub: subiculum, SuM: supramamillary nuclei, VP: ventral pallidum, VT: ventral tegmental area, IP: interpeduncular nucleus. Coronal brain images are displayed in neurological convention (left corresponds to the left side of the brain). Stimulation angles are shown on top. Stimulation frequency was 130 Hz.

**Figure 5:**
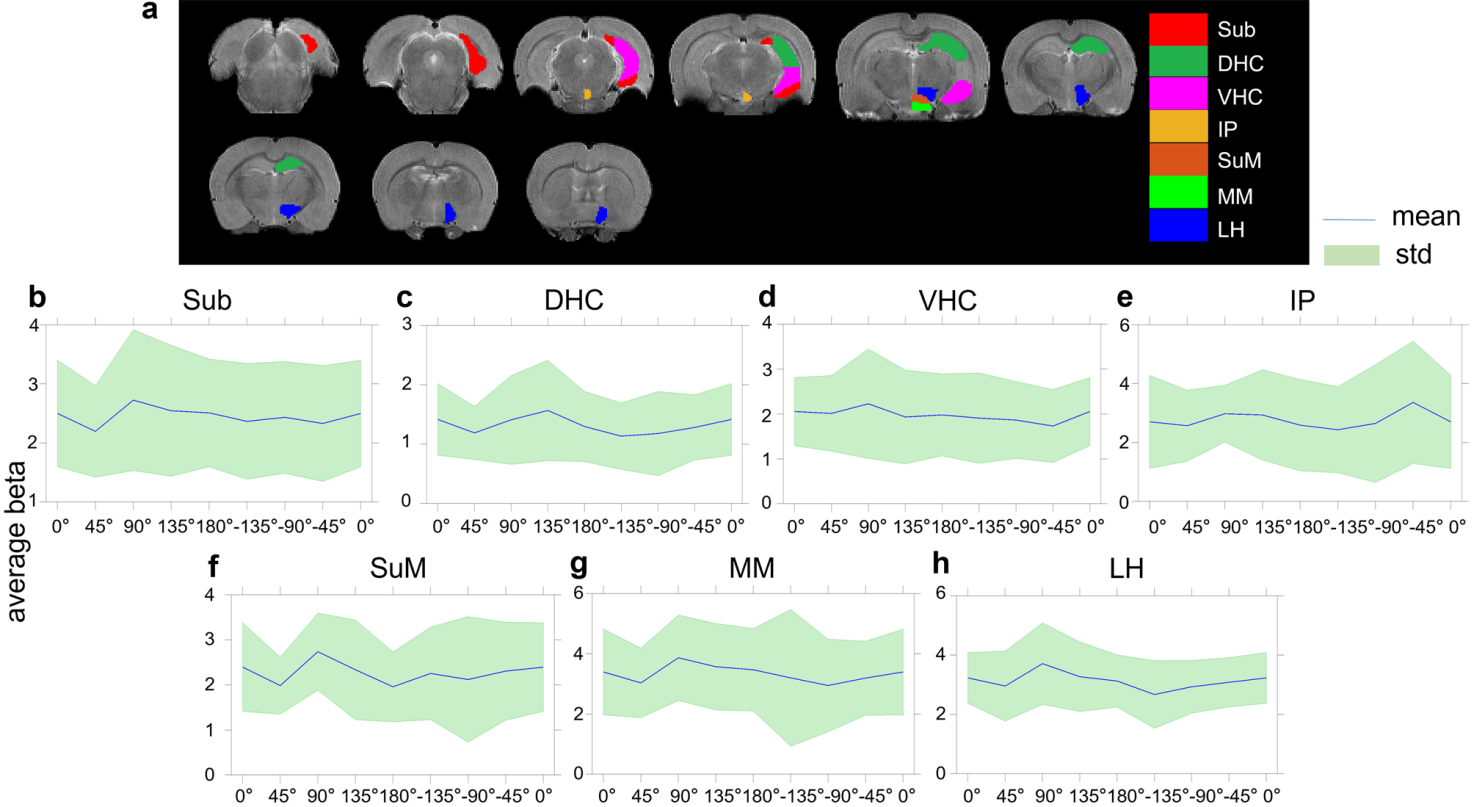
ROI analyses of OS-DBS in the MSN (n = 8). The locations of the primary ROIs are shown on T2-weighted MRI images (**a**). Average beta values are shown for the ROI in the subiculum, Sub (**b**); dorsal hippocampus, DHC (**c**); ventral hippocampus, VHC (**d**); interpeduncular nucleus, IP (**e**); supramamillary nuclei, SuM (**f**); mammillary bodies, MM (**g**); and lateral hypothalamus, LH (**h**). The blue line and green area represent the mean value and standard deviation among rats, respectively. None of the angles reached p < 0.05, corrected (linear mixed model comparisons vs − 90°, adjusted for Bonferroni multiple comparisons correction). Coronal brain images are displayed in neurological convention (left corresponds to the left side of the brain). Stimulation frequency was 130 Hz.

The typical 130 Hz frequency used in DBS clinical settings^23,25^ was found to provide robust HC response in case of MSN stimulation, and was selected for the subsequent experiments. OS-DBS of the MSN at 130 Hz activated numerous brain regions (Fig. 4 and Supplementary Fig. 4), including the primary ROIs, DHC and VHC, Sub, LH, MM, SuM and IP, and additional areas, Amg, DB, IL/PL, LS, MS, NA, SN, VP, and ventral tegmental (VT) area. These were mainly right lateralized in accordance with the verified right placement bias of the stimulation electrodes. However, no angle-dependence of the brain activity patterns were observed, neither at voxel-level in single subject (Supplementary Fig. 4) and in the group (Fig. 4), or at ROI-level (Fig. 5).

**Figure 6:**
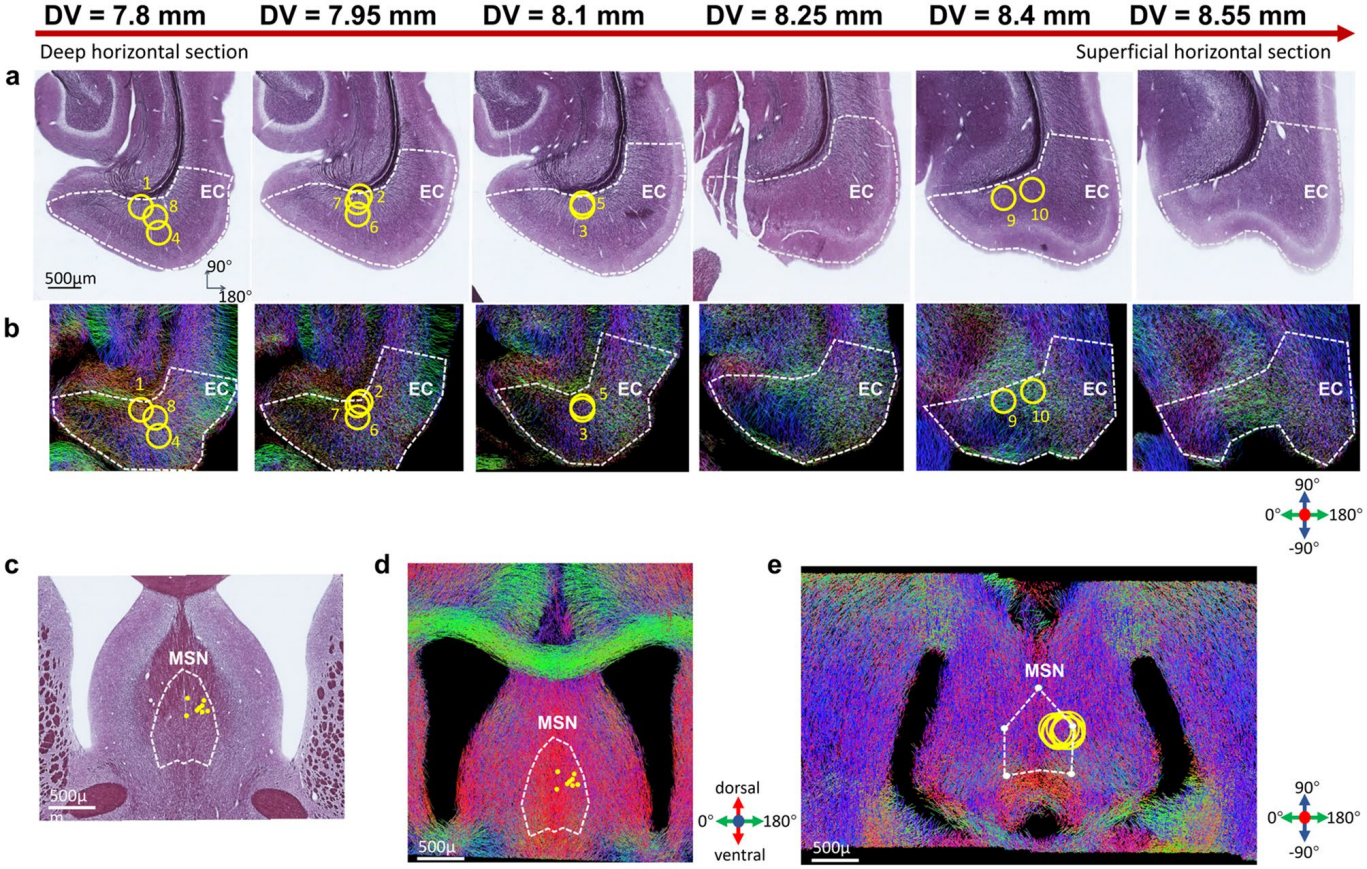
Histology and diffusion MRI tractography evaluations of EC and MSN. (**a**) Myelin staining of EC in anatomical horizontal sections corresponding to axial view in MRI. Estimated DV coordinates are displayed on top of each section. Red arrow indicates ordering from deeper to more superficial horizontal sections. (**b**) Direction-encoded color tractograms which correspond to the sections shown in a. The yellow circles indicate the location and extent of the 3-channel electrode implanted in each animal, indicated by different numbers. (**c**) Myelin staining of the MSN in coronal view; (**d**) corresponding filtered direction-encoded color tractogram in coronal view; and (**e**) tractogram in axial view. The yellow points in (**c**) and (**d**), and the circles in (**e**), indicate the center of the tips in coronal view, and the extent of the implanted electrodes in axial view, respectively. Color coding: red, dorsoventral; blue, rostrocaudal; green mediolateral.

The orientations of myelinated axons in the EC and MSN were assessed with histology (Fig. 6a,c) and diffusion MRI tractography (Fig. 6b,d,e). For more superficial EC layers, the histology shows that the axons are too sparse to identify a main orientation, while the deeper myelin sections exhibited main axon orientations ranging from about − 45° to 180°. Tractography shows complex axon distributions in the implantation sites, consistent with the observation that multiple angles at times provided comparably strong fMRI clusters in individual rats (Supplementary Fig. 3). Despite the observed complex axon distributions in each rat, − 45° appeared to be one of the main axon orientations around the target position in most rats (Supplementary Fig. 1). Notably, OS-DBS stimulations at − 45° provided distinctively bigger fMRI clusters in connected areas to the EC, including HC, Sub, Amg, and PrC (Fig. 2).

For the MSN, the main axonal orientation was dorsal–ventral, i.e., along the electrode itself. Such observation explains the lack of orientation selectivity observed for OS-DBS of MSN, as in fact the reorientation of the stimulation angle was achieved on the plane encompassing the tips of the 3-channel electrode perpendicular to the major fiber orientation.

Finally, we report individual experimental currents and electrode locations in Supplementary Fig. 1 for EC implantation, and in Supplementary Fig. 2 for MSN implantation, while all individual activation maps are documented in Supplementary Figs. 3 and 4. In case of EC implantation for which stimulation angle dependence was observed, voltage field distributions obtained with COMSOL at representative stimulation angles are shown on myelin-stained sections encompassing the implantation site for each rat (Supplementary Fig. 1a). Electrode tip locations are shown on tractograms sections (Supplementary Fig. 1b for EC in axial view; Supplementary Fig. 2a,c for MSN in axial and coronal view, respectively). Electrode tips are also shown on coronal MRI images overlapping with the atlas^36^ in Supplementary Fig. 1d for EC and Supplementary Fig. 2d for MSN, and on the atlas sections alone (panel c and e of Supplementary Fig. 1 for EC in axial and coronal view, respectively; panel b and e of Supplementary Fig. 2 for MSN in axial and coronal view, respectively).

## Discussion

In this work, we demonstrated that varying the stimulation angle in OS-DBS can be exploited to modulate the activity of the networks connected to the EC and MSN. Maximal fMRI responses of downstream areas to the EC generally occurred at stimulation angles of − 45°, which was one of the main axonal directions of the EC nerve fiber efferents observed in deep horizontal sections (i.e., further from the skull, Fig. 6a), and 180°. The ventral region of the hippocampus, which is closer to the EC in rodents, was primarily activated during OS-DBS of the EC, and showed a stimulation angle effect. On the other hand, for the MSN group, the fMRI responses in HC and brain areas connected to the MSN did not vary significantly between different stimulation angles with the planar 3-channel electrode used here. Being a midline region, MSN is a particularly promising target for stimulation. Brain activations during MSN stimulation were indeed very robust and covered the entire HC, in agreement with findings that MSN optogenetic stimulation modulates electrical activity in the rostral to caudal extent of the HC^21^. Note that, in these data, activations were more prominent in one hemisphere because the electrodes were often slightly offset to the right side of the MSN.

This study shows the feasibility of OS-DBS for stimulating areas relevant to Alzheimer’s disease, setting the framework for further technological developments and applications of multielectrode arrays to substantiate the potential of our findings as a direction for treatment therapy in Alzheimer’s disease. The flexibility offered by OS-DBS in enhancing the activation of a specific circuitry by targeting the axonal directions of interest within the stimulation site may be beneficial to reduce stimulation-related side effects while enhancing therapeutic effects in chronic stimulation settings.

Although statistically significant group-level modulatory effects induced by the stimulation angle were observed in our group of 10 rats implanted in the EC, large variability of activation patterns as a function of the stimulation angle was also observed. As the EC is comprised of five cortical layers^38^, with different layers projecting differently to the HC, the location of the electrode relative to such layers has the potential to significantly affect the outcomes of DBS. Specifically, neurons in layer II project to the dentate gyrus and CA3, while neurons in layer III have projections to CA1 and subiculum^38^. The deep layers, especially layer V, receive projections from the HC^39^, and project to EC superficial layers and extrahippocampal brain structures^38^. Moreover, some axons project to extrahippocampal brain structures like amygdala, some axons project to the layer II of EC then to CA1 and dentate gyrus, some axons project to layer III then to CA3. Based on such considerations, it is thus conceivable that OS-DBS leads to somewhat different activation patterns when stimulating the deeper layer as compared to the superficial layers, a prediction that may explain the large inter-subject variability of modulatory effects induced by OS-DBS as observed in our study. In addition, it should be kept in mind that the large electrode size could lead to some axons in deeper layers to be stimulated when stimulating the superficial layers. Importantly, it should be noted that the convention used for assigning “superficial” and “deep” horizontal myelin staining sections in Fig. 6a generally follows the convention of superficial and deep cortical layers^38^, but a precise delineation of the cortical layers as defined in^38^ for each horizontal section cannot be achieved in the absence of a complete 3D structure determination of the EC. Therefore, a one-to-one assignment of electrode location in each rat within the exact cortical layer cannot be robustly accomplished. The complex nature of the EC architecture was confirmed by the complementary information obtained from the tractograms (Fig. 6b), which showed a range of orientations of the major tracts according to the slice position from deep to superficial layers. Worth noting is that histology provides information only about myelinated axons, while diffusion MRI tractograms provide information about all the structures affecting water molecule movement.

Absence of brain activations was observed at − 90° in the group analysis and, at single subject level, in 6 out of 10 rats implanted in EC. This was likely because at − 90° the negative pole of the stimulation vector was quite close to the edge of the brain where the density of the axons is lower. OS-DBS at − 45° resulted in large spread of activation both in the group analysis and in the majority of rats at single subject level, which was in agreement with main axonal orientations in the deep layers indicated in Fig. 6. At 135°, which is opposite to the − 45° orientation, the fMRI response was minimal. This could be as a result of partial anodic block generated by the anodic lobe of the dipole stimulation^40^.

Several other brain structures also exhibited strong fMRI responses when stimulating the EC, including the amygdala and IL/PL, which are involved in memory consolidation^41^. The amygdala receives strong EC projections, mainly from lateral entorhinal cortex (LEC)^42–44^, and further connects with the ventral (temporal) CA1 subdivision of the HC, and perirhinal cortex^45^. The IL/PL also receives major projections from LEC^46^. Further, we observed activation in the nucleus accumbens, which receives direct EC projections^47^. In addition, strong activation was seen in piriform cortex and insula, both of which receive direct projections from LEC^38^.

OS-DBS of the MSN elicited strong fMRI responses in the dorsal and ventral HC, subiculum, amygdala, and medial frontal cortex (IL/PL). In addition, MSN stimulation activated the mamillary and supramamillary nuclei that receive hippocampal projections through the fornix. For MSN, the main orientation of myelinated axons was dorsal–ventral (Fig. 6d). Therefore, varying the main electrode field direction in an axial plane did not result in significant differences in activation patterns (Fig. 4, Supplementary Fig. 4). Future studies are thus required for achieving OS-DBS along a dorsal–ventral orientation, for which electrodes with minimally four independently driven channels distributed in 3D rather than on a plane are required for re-orienting the electric field gradients in space.

The MRI findings most likely reflect not only contributions from orientation selective stimulation of axons within the target, but also from the specific location of the effective cathode during OS-DBS. For instance, the activation of the HC could be induced because of proximity of the effective cathode to the angular bundle and subiculum, where fibers from the EC converge with primary orientations of 0° and 90° and project to the hippocampus. This conceptual framework is supported by the observation that strong HC activation occurs at 0°, although is supported to a less extent at 90° where we do not see a response as strong as at 0°. The substrates that mediate the activation of other structures at angles of 180° and -135° are less obvious, but at these angles, the stimulation is directed towards the entorhinal cortex, including its deep layers that project to cortical areas including those activated at these angles.

Several limitations of the study should be noted. First, the sample size of the study was relatively small (10 animals for EC implantation and 8 for MSN implantation). To mitigate this limitation, we used strict threshold criteria in the generation of group-based activation maps, and further document the individual single subject results. Moreover, the relatively large size of the 3-channel electrode, along with the high stimulus intensity used in these acute DBS study, imply that different OS-DBS angles may stimulate surrounding areas of the selected targets thus leading to widespread activations beyond those of the targeted circuitry. However, on the other hand, a small contact size could lead to tissue damage because of the high energy deposition per unit time. Higher electrode counts and different electrode configuration are also desired to achieve re-orientation of the electrical field in space rather than on a plane like that exemplified in the present study. Despite the use of the stereotactic system and despite all efforts had been allocated to ensure precise implantation, implantation inaccuracies can still take place, potentially introducing variability in the activation patterns even within the same group of animals. The electrodes may also be inserted into the tissue in slightly twisted manner, so the actual determination of the stimulation angles may be compromised across the animals thus preventing accurate group analyses as a function of the stimulation angle. Moreover, the characterization of fiber orientations based on tractography and myelin staining is highly informative, but it has also limitations. We can extract fiber orientation from tractograms reconstructed at 150-µm thick slice. As a grey matter area, the presence of fiber bundles in EC is low as observed in myelin staining, and the tractograms reflect all orientations of the structures contained in that relative thick slab of tissue, including myelinated, non-myelinated axons or dendrites. For a more accurate estimation of the angular differences between OS-DBS experiments, higher resolution diffusion MRI, other histological approaches, e.g. 3-dimensional confocal microscopy, and determination of electrode locations should ideally be conducted in each individual animal, rather than using a reference brain as it was done in the present study. Yet, the a priori knowledge of axon orientations is helpful for narrowing the parameter space of stimulations angles and for the validation purposes, but it is not intrinsically required for exploiting the OS-DBS framework. Indeed, the flexibility offered by OS-DBS to vary the stimulation angle, together with the opportunity to measure whole-brain activity with fMRI during OS-DBS, allows to maximize in real-time the activation of connected downstream brain areas also when the direction of axons is not precisely known in the individual subject, and even in presence of minor electrode placement inaccuracies.

The 1–2 mA current used in this study, higher than typically utilized clinically, was motivated by the need of achieving robust fMRI responses. It should be noted that anesthesia itself most likely challenges the efficacy of electrical stimulation for inducing fMRI responses. Moreover, the implantation of the DBS electrode results in acute edema around and along the lead, causing a decrease in the amount of current spreading into the tissue and a decrease in extracellular signal amplitude^48^ that may reflect in smaller fMRI contrast. Worth noting is that the presence of edema can also compromise the selectivity of stimulation given the current spreading into the surrounding areas beyond the target. Notably, our preliminary results in awake animals with chronically implanted DBS electrodes (unpublished), where anesthesia does not play a role and the edema around the electrode is supposed to be resolved, suggest that the level of current needed for achieving detectable fMRI responses is up to one order of magnitude lower than in anesthetized rats. Overall, the high currents used in anesthetized animals may cause tissue damage, and even the MRI pulse sequences can induce unwanted currents in DBS leads because of RF heating of the tip of the electrode^49,50^. Histological evaluations were not conducted in this work, however visual inspection of the anatomical images at the end of the experiments suggested no tissue damage, consistent with other DBS studies in rodents when using balanced bipolar pulses with similar level of current 1–2 mA^30–32,34,51,52^.

The use of anesthesia in the current study allowed acquiring fMRI responses to stimulation at several angles for investigating the effects of OS-DBS. However, awake animals cannot remain comfortable in the scanner for more than ∼45 min. Therefore, OS-DBS study designs will require fewer stimulation angles and/or shorter stimulation trains to achieve tolerable scan times, despite the animal is expected to tolerate well long stimulation trains because lower currents can be used in the awake state. Worth noting, while lower currents will benefit safety and stimulus tolerance, they may potentially impair stimulus efficacy and selectivity. In fact, previous neuronal modelling results^30^ showed clear OS-DBS effects with 1–2 mA currents and similar electrode design used in this study, but lower currents and different electrode configurations were not explicitly investigated, including different inter-contact distances or higher channel counts. Overall, dedicated modeling and experimental investigations will need to be undertaken to evaluate electrode designs, stimulus parameters, and MRI protocols with the goal of optimizing OS-DBS in awake animals. Finally, for EC stimulation, in the present study we used a 20 Hz stimulation frequency, which is a classic kindling paradigm for inducing epilepsy^53^. Therefore, future studies may be focused on circumventing kindling by utilizing high inhibitory frequency of stimulation, 100 Hz and higher. High frequency stimulation (HFS) is used in treating Parkinson’s disease patients with DBS targeted to globus pallidus (GP) or STN. In both cases, DBS at these frequencies decrease firing of talamocortical neurons. The exact underlying neural mechanisms are still somewhat unsettled, but the general idea as reviewed by Liu and colleagues^54^ is that HFS suppresses firing at the nearby neuronal somata, possibly via a depolarization block, while nearby axons are stimulated at the applied frequency. In case of basal ganglia, the axons stimulated at these high frequencies are the GABAergic axons of GP neurons, which inhibit STN or thalamic glutamatergic neurons. In case of entorhinal cortex stimulation, similarly GABAergic neurons impinging on layer 2 projection neurons may get activated. It has been shown that prolonged (> 10 min) HFS at EC does not evoke seizures in mice^10^. Future studies are also warranted in larger animal models to better resemble clinical settings.

In conclusion, we conducted the first in vivo investigations of OS-DBS of the EC and MSN in rats. Such stimulation targets are potential DBS targets in Alzheimer’s disease given their involvement in memory and cognition and their association with induction of neurogenesis in the dentate gyrus of the hippocampal formation. OS-DBS of the EC modulated brain activity in the HC which is crucial for spatial and episodic memory, in the amygdala which is involved with emotional memory modulation, in the medial frontal cortex (IL/PL) involved with several cognitive functions. Strongest activation of HC, subiculum, amygdala, and perirhinal and piriform cortex was achieved when stimulating the EC at − 45° at the group level, in general agreement with the outcomes provided by histological and MRI tractography assessments. No significant dependence of the activation on OS-DBS of the MSN had been detected, which was attributed to the orthogonal orientation of axonal tracks relatively to the stimulation plane of the implanted 3-channel electrode. Further studies with multichannel electrode with higher channel count, and in larger animals using commercial electrodes, are required for substantiating the potential of OS-DBS as a treatment therapy for Alzheimer’s disease. Since OS-DBS can be implemented with available commercial electrodes^33^, and fMRI can be safely used during DBS^55^, the conceptual framework of combining OS-DBS with fMRI for manipulating and monitoring brain network activity, respectively, holds great promise for translation to clinical settings.

## Methods

### Surgical procedures and electrode implantations

The study was carried out in compliance with the ARRIVE guidelines, and all surgical and experimental procedures were approved by the Institutional Animal Care and Use Committee (IACUC) of the University of Minnesota. Sprague–Dawley rats (Envigo; Madison, WI, USA; male, 252–340 g, n = 12 for EC; n = 8 for MSN) were housed in pairs in a temperature and humidity-controlled vivarium with a 12-h light–dark cycle with ad libitum diet. Rats were initially anesthetized using isoflurane for the duration of the implantation (5% for induction, 1–3% during surgery) with O_2_/N_2_O (30%/70%) carrier gas. The respiration rate was monitored using a plastic pressure sensor during the whole study. The temperature was monitored using a rectal thermometer and maintained at 37 °C with a heating pad during the surgery, heated water circulation and heated air during MRI. After the electrode implantation, the anesthesia was changed to urethane (1.5 g/kg) with four consecutive injections 15 min apart while gradually lowering the isoflurane level to reach 0%. Urethane was used to replace isoflurane as it enables a strong fMRI response^56^ and maintains normal blood gas levels in spontaneously breathing rats^57^. Each animal was placed on a stereotactic frame and a craniotomy was made by drilling through the skull on the right hemisphere. A tripolar lead composed of a twisted set of three polyimide-insulated tungsten wires (PlasticsOne, MS333T/2C-A/SP; Roanoke, VA, USA) with tip-contact diameters of 127 µm, and including the insulation layer 157 µm (total diameter of three electrode bundle ~ 350 µm), were implanted in the right EC and MSN. For EC, the targeted coordinates were: anterior–posterior (AP) = − 6.85 mm, medio-lateral (ML) = − 5 mm, and dorsal–ventral (DV) = 8 mm; for MSN, they were AP =  + 0.1 mm, ML =  + 0.4 mm, and DV = 5.8 mm based on the rat atlas^36^. Three to four drops of 2% lidocaine were administered before the incision of the scalp for localized anesthesia, and before cauterizing vessels of the scalp and skull. The remaining hole in the skull around the electrode was filled with gelatin foam (SPONGOSTAN, Søborg, Denmark), then covered with dental acrylic (Lang Dental, Jet Acrylic, Wheeling, IL, USA). Finally, an Ag/AgCl wire (5 cm long, 0.5 mm diameter) acting as a ground electrode, was inserted below the skin of the neck.

### MR data acquisition and stimulation paradigms

The MRI scans were conducted in a 9.4-T 31-cm horizontal-bore magnet equipped with Agilent Direct DRIVE console (Palo Alto, CA, USA) using a quadrature radio frequency coil with full rat brain coverage. Shimming was performed inside an approximate cerebrum sized voxel (10 × 10 × 6.5 mm^3^) using a field mapping based shimming protocol included in the Agilent VNMRJ 4.0 package.

Before fMRI, high resolution anatomical images in coronal view were taken using fast spin-echo (FSE) pulse sequence: repetition time TR = 3 s, effective echo time TE = 48 ms, Echo train length = 4, matrix size = 192 × 192, field of view FOV = 32 × 32 mm^2^, slice thickness = 1 mm and number of slices = 15 from rat’s AP − 4.5 mm to AP 9.5 mm, number of averages = 4. These T_2_-weighted FSE images were used to verify the electrode location and estimate the DV of the electrode tip by overlapping the MRI images with the rat brain atlas^36^ (Supplementary Figs. 1, 2).

MB-SWIFT^58^ was used for fMRI to minimize susceptibility artefacts originating from the presence of the electrodes as well as motion artefacts. MB-SWIFT is a 3D radial free induction decay (FID) based pulse sequence with virtually zero acquisition delay and high excitation and recording bandwidths. The functional contrast of MB-SWIFT has been recently investigated and attributed to blood in-flow effect providing similar results with Blood Oxygen Level Dependent (BOLD) contrast^34^. The following parameters were used in MB-SWIFT fMRI: TR = 0.97 ms, 3094 spokes per volume, resulting in temporal resolution of 3 s, BW = 192 kHz, matrix size = 64^3^, FOV = 3.2 × 3.2 × 6.4 cm and flip angle = 5°. The center of FOV was set to -0.5 mm, the same as FSE image to ensure an overlap of FSE and MB-SWIFT images. Excitation was performed with a chirp pulse gapped into four 2.6 µs sub-pulses^58^. Two-fold oversampling was used during acquisition in the gaps of 32/BW duration. The post-correlation FID consisted of 32 points.

The stimulation paradigm for each stimulation angle consisted of 60 s of rest and 18 s of stimulation, repeated three times and ending in a rest period, for a total of about 5 min of acquisition time. Stimulation was applied using symmetric biphasic square pulses with 200 µs duration per phase, delivered with an 8-channel stimulus generator (STG-4008-16 mA Multi Channel Systems, Warner Instruments LLC, Hamden, CT, USA) in current controlled mode, driven by the company’s software with input waveform generated in MATLAB 2017b (MathWorks; Natick, MA, USA). For stimulation of EC and MSN the frequencies of 20 Hz and 130 Hz were used, respectively. For the OS-DBS, the current amplitudes I_1,2,3_ for each one of three electrode were calculated based on sinusoidal functions with phase offsets of $120^\circ$^30^ according to

1 $$\begin{aligned} {I}_{1} & ={I}_{0}cos(\mathrm{\varnothing }+120^\circ ) \\{I}_{2} & ={I}_{0}cos(\mathrm{\varnothing }) \\ {I}_{3}&={I}_{0}cos\left(\mathrm{\varnothing }-120^\circ \right),\end{aligned}$$

where ${I}_{\mathrm{1,2},3}$ are the current amplitudes contacts 1 to 3, ${I}_{0}$ is the stimulation current amplitude and $\mathrm{\varnothing }$ governs the stimulation angle. The stimulation current in each animal was set based on initial fMRI scans by driving the tripolar electrode as monopolar to identify the current level giving a non-artefactual, robust fMRI response in the hippocampus. The orientation of the electric field was set to varying angles between 0° to 315° (i.e., − 45°) with steps of 45°, for a total of 8 stimulation angles. The angles were set such that 0°/180° corresponds to the medial–lateral direction while − 90°/90° corresponds to the rostral-caudal direction, respectively (as indicated on top of Figs. 2 and 4). The order of angles was randomized across animals, as shown in Supplementary Figs. 3 and 4.

For illustration purposes, we also calculated with COMSOL 5.4 (COMSOL, Stockholm, Sweden) the voltage field distributions of OS-DBS at representative angles by considering the actual size of the 3-channel electrode, as shown in Fig. 1 and in Supplementary Fig. 1.

### MRI data processing and statistical analysis

MB-SWIFT images were reconstructed by gridding with three iterations FISTA algorithm^59^. The resulting data were analyzed in SPM8 (http://www.fil.ion.ucl.ac.uk/spm), MATLAB 2017b (The MathWorks, Inc., Natick, MA, http://www.mathworks.com). Prior to SPM8 analysis, functional data were first corrected for motion artefacts, then co-registered to the corresponding anatomical data and finally normalized to an animal without an electrode outside the fMRI group based on FSE images using the transformation of the anatomical data as done previously^32^. Then data was smoothed with a [1 1 1] pixel FWHM Gaussian kernel. The single-subject analysis was computed using a general linear model that consisted of a block design model convolved with a first-order gamma hemodynamic response function of 15 s duration^60^. A 1000 s cut-off high-pass filter was applied on the functional data in the temporal domain. A correction for serial auto-correlation in SPM was also applied using a first-order auto-regressive model applied to the residuals.

At first, the level threshold for statistical significance for positive and negative activations in individual rats was set to family wise corrected (FWE) with p < 0.05. The beta maps were then used for the group-based statistical analysis by applying a one-way within-subject ANOVA model with the stimulation angle defined as a factor (8 levels, one for each angle from 0° to 315° with 45° per step) to get the maps of main effects. Maps of main effects were finally computed after applying a statistical threshold of p ≤ 0.05 (FWE). A brain mask was also applied to the group main effects.

To quantify the differences of the fMRI responses to different OS-DBS angles, we applied a linear mixed model with fixed effects for angle to the beta-values averaged in anatomically defined regions of interest (ROIs) relevant to memory and cognitive functions, ipsilateral to the stimulation site (right side), and drawn manually with Aedes (aedes.uef.fi) based on The Rat Brain in Stereotaxic Coordinates (6th Edition)^36^. The ROIs considered for OS-DBS of the EC included the hippocampus (HC) and the subiculum (Sub), i.e., areas relevant to memory and cognitive function, and areas connected to the EC, namely the perirhinal cortex (PrC), the piriform cortex (Pir), the amygdala (Amg) and the insula (Ins). The ROIs considered for OS-DBS of the MSN included the HC, the Sub, and areas connected to the MSN, namely the interpeduncular nucleus (IP), the lateral hypothalamus (LH), the mammillary bodies (MM) and supramamillary nuclei (SuM). Pair-wise comparisons of different angles were performed, and the contrast between each pair of angles was tested against a two-sided null hypothesis. Bonferroni correction was performed separately for the comparison of each angle to the other 7 angles, and level of significance was set at p = 0.05 Bonferroni corrected (i.e., p = 0.007 uncorrected). Descriptive statistics measures are indicated as Mean ± SD.

### Diffusion MRI tractography and histology

The assessment of axon orientations within EC and MSN was achieved by diffusion MRI and histology as in our previous studies^30,32^. For this work, three adult male Sprague–Dawley rats outside the DBS group (300 g, Harlan Netherlands B.V., Horst, Netherlands) were used. The rats were housed in an individual cage and kept under a normal 12 h light/12 h dark cycle with constant temperature (22 ± 1 °C) and humidity (50–60%). Water and food were available ad libitum. This animal procedure was approved by the Animal Ethics Committee of the Provincial Government of Southern Finland and carried out in accordance with the guidelines of the European Community Council Directives 2010/63/EEC.

After the perfusion, the rat brain was scanned in a vertical 9.4 T/89 mm magnet (Oxford Instruments PLC, Abingdon, UK) interfaced with a DirectDrive console (Varian Inc., Palo Alto, CA, USA) using a quadrature volume RF-coil (Ø = 20 mm; Rapid Biomedical GmbH, Rimpar, Germany) as a transceiver. The data were acquired using a 3D spin-echo-echo-planar-imaging sequence using the following parameters: TR/TE = 800/35 ms, echo spacing = 0.584 ms, number of shots = 4, BW = 250 kHz, number of averages = 2, FOV = 16.2 × 12.0 × 14.1 mm^3^, matrix size = 96 × 108 × 160, spatial resolution = 150 × 150 × 150 μm^3^, number of diffusion directions = 60 for each b value, of 1500 and 3000 s/mm^2^, number of minimally diffusion weighted images = 4, diffusion gradient amplitude = 3.5 mT/cm and duration (δ)/separation (Δ) = 6/11.50 ms, and acquisition time = 20 h 48 min.

The data was pre-processed using MRtrix3^61^ consisting of denoising^62^ and simultaneous motion and residual eddy current induced geometric distortion correction. In order to increase anatomical contrast, the data was upsampled by a factor of two using cubic interpolation. Constrained spherical deconvolution (CSD) was used to resolve crossing-fibers by estimating the fiber orientation distribution at each voxel. The response function for white and gray matter and free water was calculated with Dhollander algorithm within MRtrix3^61^. A CSD-based whole-brain probabilistic tractography dataset of 30 million streamlines were computed. For the tractogram, iFOD2 algorithm was used^63^ with a step-size of 0.037 mm, maximum angle of 45° between steps, with a minimum and maximum streamline length of 2 mm and 5 mm, respectively. The resultant tracks were filtered with spherical-deconvolution informed filtering of tractograms^64^ in the regions of interest to five hundred thousand streamlines.

Coronal and horizontal sections from two rat brains stained with gold chloride for myelin^65^ were used to verify the orientation of the myelinated axons in EC and MSN. Photomicrographs were taken with a light microscope (Zeiss Axio Imager2, White Plains, NY, USA) equipped with a digital camera (Zeiss Axiocam color 506, White Plains, NY, USA). The orientation of the axons was estimated using ImageJ software (1.47v, NIH, USA).

## Supplementary Information

Supplementary Figures.

## Data Availability

The datasets generated during and/or analyzed during the current study are available from the corresponding author on reasonable request.
