# Supplementary material for: Orientation selective DBS of entorhinal cortex and medial septal nucleus modulates activity of rat brain areas involved in memory and cognition
Source: Sci Rep. 2022 May 20;12:8565. doi: 10.1038/s41598-022-12383-2 (PMC9122972; doi:10.1038/s41598-022-12383-2)
Supplement: Supplementary file 1 — Supplementary Figures. [file 41598_2022_12383_MOESM1_ESM.pdf]

SUPPLEMENTARY MATERIAL

Orientation-Selective DBS of Entorhinal Cortex and Medial Septal Nucleus modulates activity of rat brain areas involved in memory and cognition

Lin Wu[1], Antonietta Canna[1,2], Omar Narvaez[3], Jun Ma[4], Sheng Sang[1], Lauri J. Lehto[1], Alejandra Sierra[3], Heikki Tanila[3], Yuan Zhang[5], Olli Gröhn[3], Walter C. Low[4], Pavel Filip[1,6], Silvia Mangia[1#] and Shalom Michaeli[1#*]

1. Center for Magnetic Resonance Research, University of Minnesota, Minneapolis, MN, USA
2. University of Campania "Luigi Vanvitelli", Naples, Italy
3. A. I. Virtanen Institute for Molecular Sciences, University of Eastern Finland, Kuopio, Finland
4. Department of Neurosurgery, University of Minnesota, Minneapolis, USA
5. Division of Biostatistics, School of Public Health, University of Minnesota, Minneapolis, MN, USA
6. Department of Neurology, Charles University, First Faculty of Medicine and General University Hospital, Prague, Czech Republic

# shared senior authorship due to equal contribution

*Contact Information:

Shalom Michaeli, PhD
University of Minnesota, Radiology Department
Center for MR Research 2021 6th St. SE
Minneapolis, MN 55455
Phone: (612)-626-2001
E-mail: micha042@umn.edu
FAX: (612)-626-2004

Supplementary Figure 1: electrode location in EC

a) Myelin stained section in horizontal view estimated to encompass the tip of the electrode. The voltage field distribution of a stimulation angle with strong fMRI responses in the hippocampus is also shown.
b) Tractogram in horizontal view. The yellow circle shows the size and location of the 3-electrode bundle.
c) Estimated location of the electrode on an horizontal view from the atlas by Paxinos and Watson (2007) “The Rat Brain in Stereotaxic Coordinates (6th Edition)”. Blue circles indicate the size and location of the 3-channel bundle.
d) MRI image in coronal view overlaid on the atlas by Paxinos and Watson (2007) “The Rat Brain in Stereotaxic Coordinates (6th Edition)”. The red point indicates the tip of the electrode. The MRI image is displayed in neurological convention (left side of the image corresponds to the left side of the brain).
e) Estimated location of the electrode on the same coronal view of the atlas by Paxinos and Watson (2007) “The Rat Brain in Stereotaxic Coordinates (6th Edition)” as in d). The red point indicates the tip of electrode.

Rat's number and used current amplitude are indicated on the top left. The dorsal-ventral (DV) coordinate of the electrode tip is indicated on the bottom, right. Atlas images by Paxinos and Watson (2007) “The Rat Brain in Stereotaxic Coordinates (6th Edition)” are taken with permission.

EC group

Rat 1, 1.5 mA

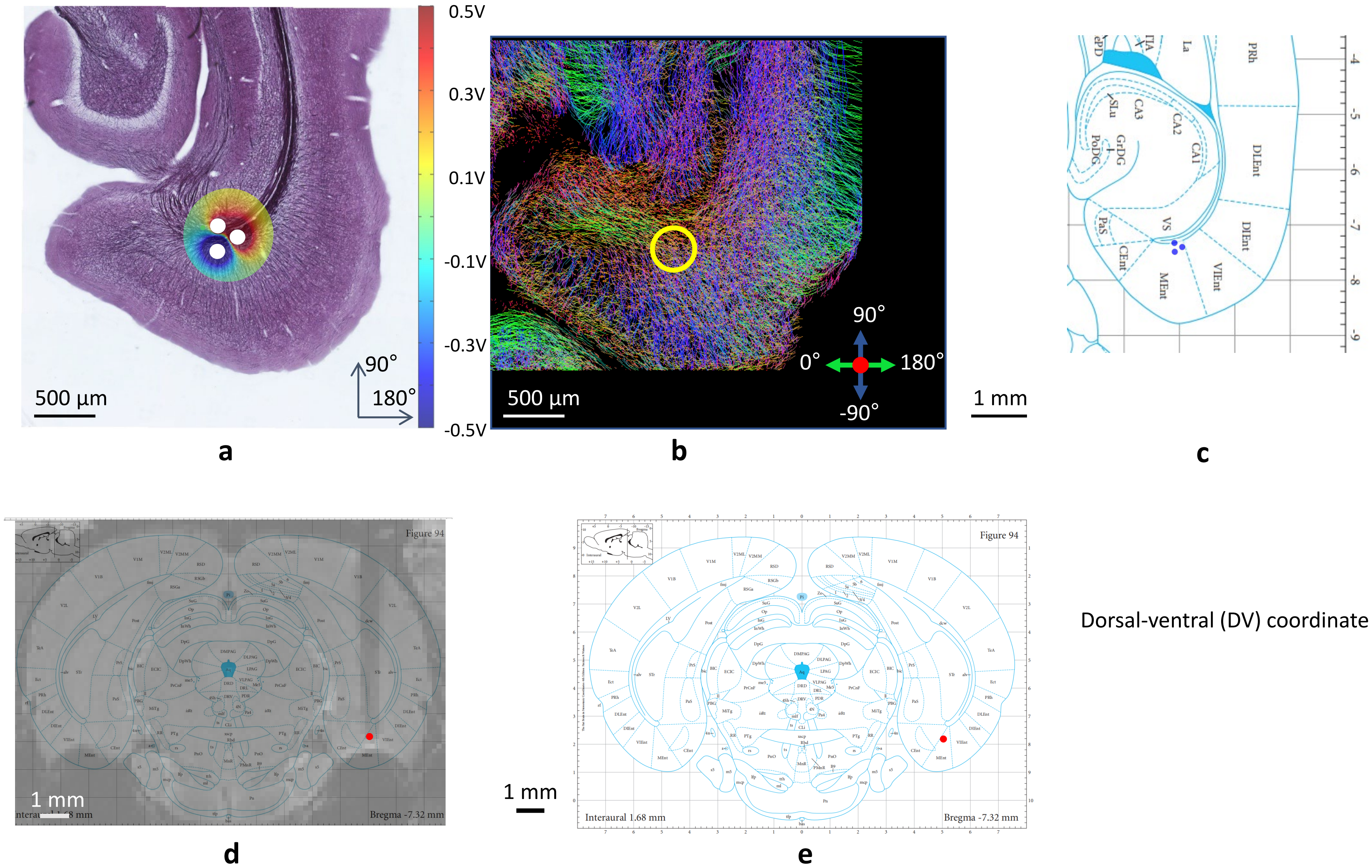

Dorsal-ventral (DV) coordinate = 7.8 mm

EC group

Rat 2, 1.0 mA

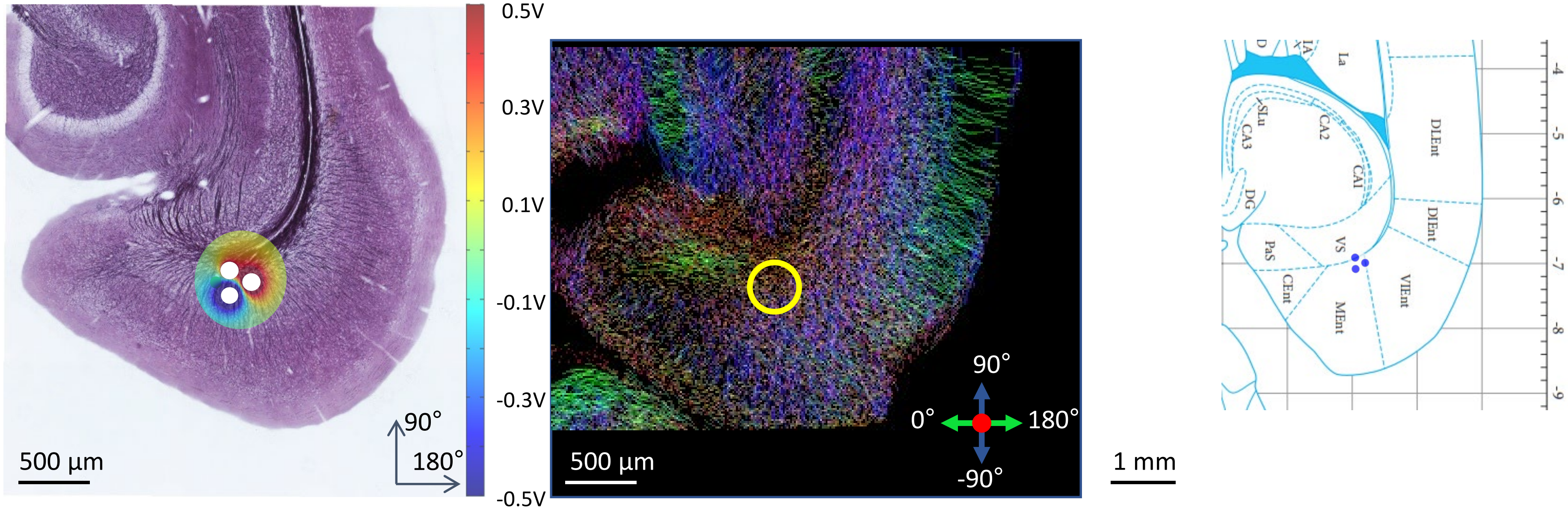

a b c

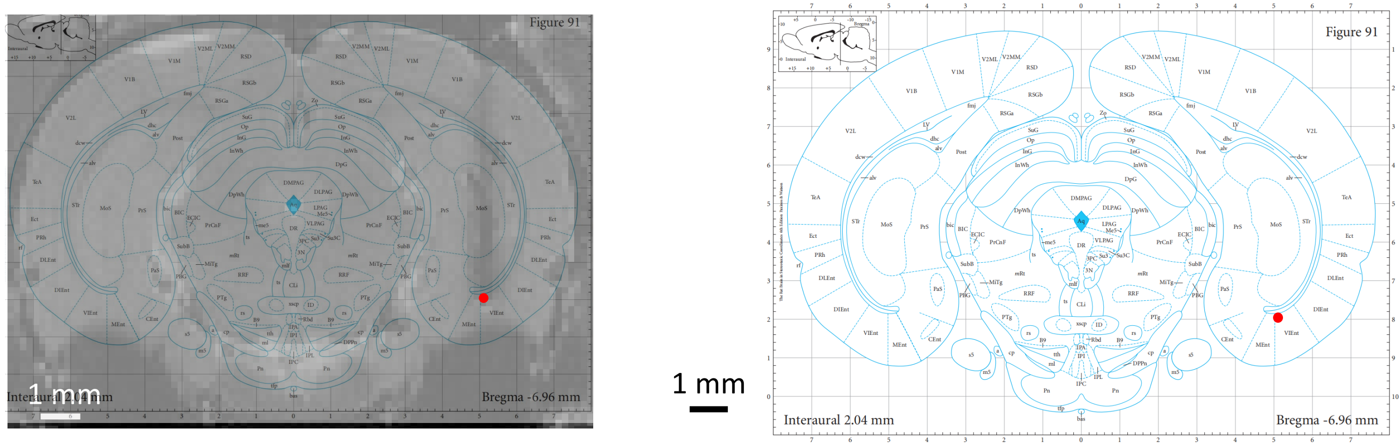

d e

Dorsal-ventral (DV) coordinate = 8 mm

EC group

Rat 3, 1.25 mA

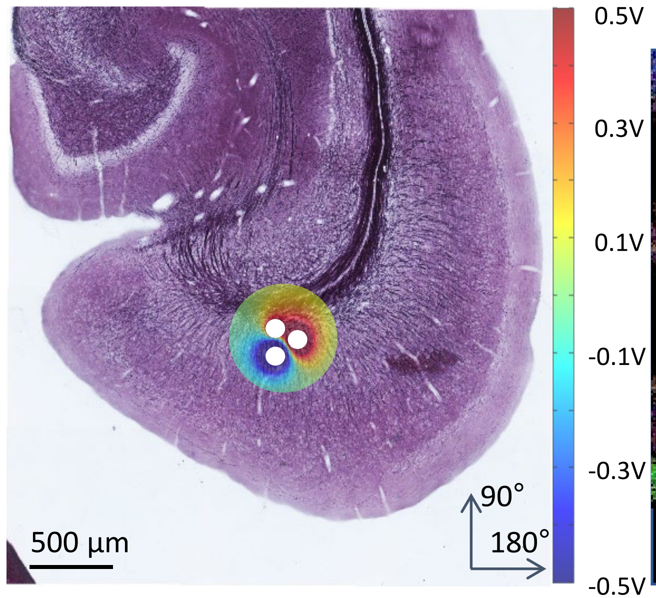

a

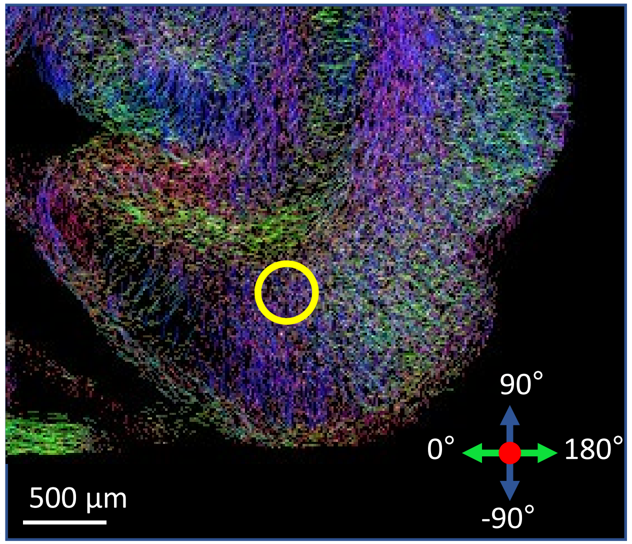

b

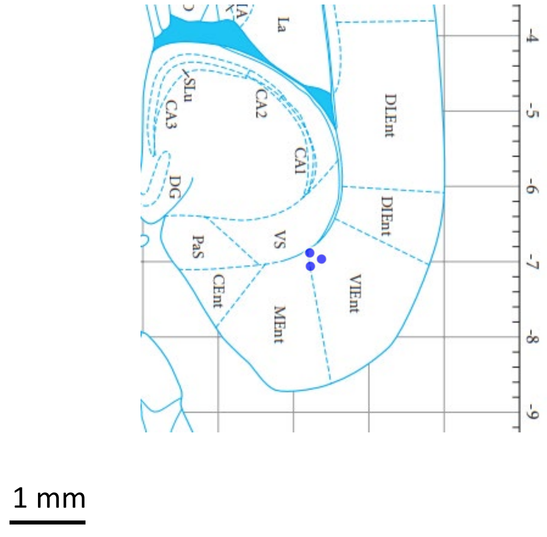

c

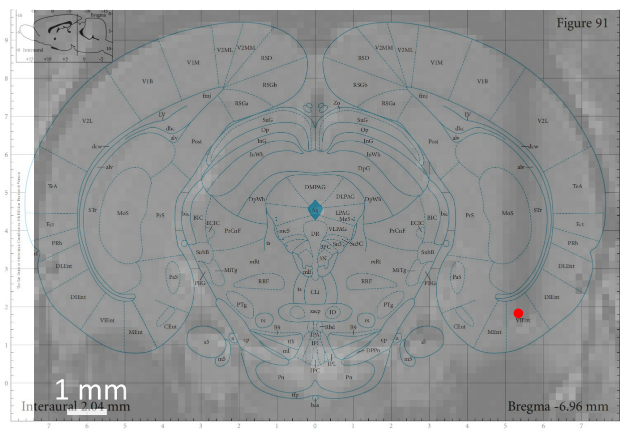

d

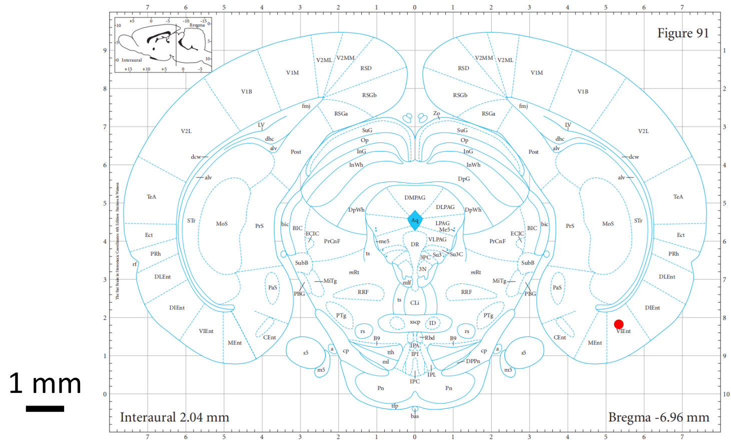

e

Dorsal-ventral (DV) coordinate = 8.17 mm

EC group

Rat 4, 1.75 mA

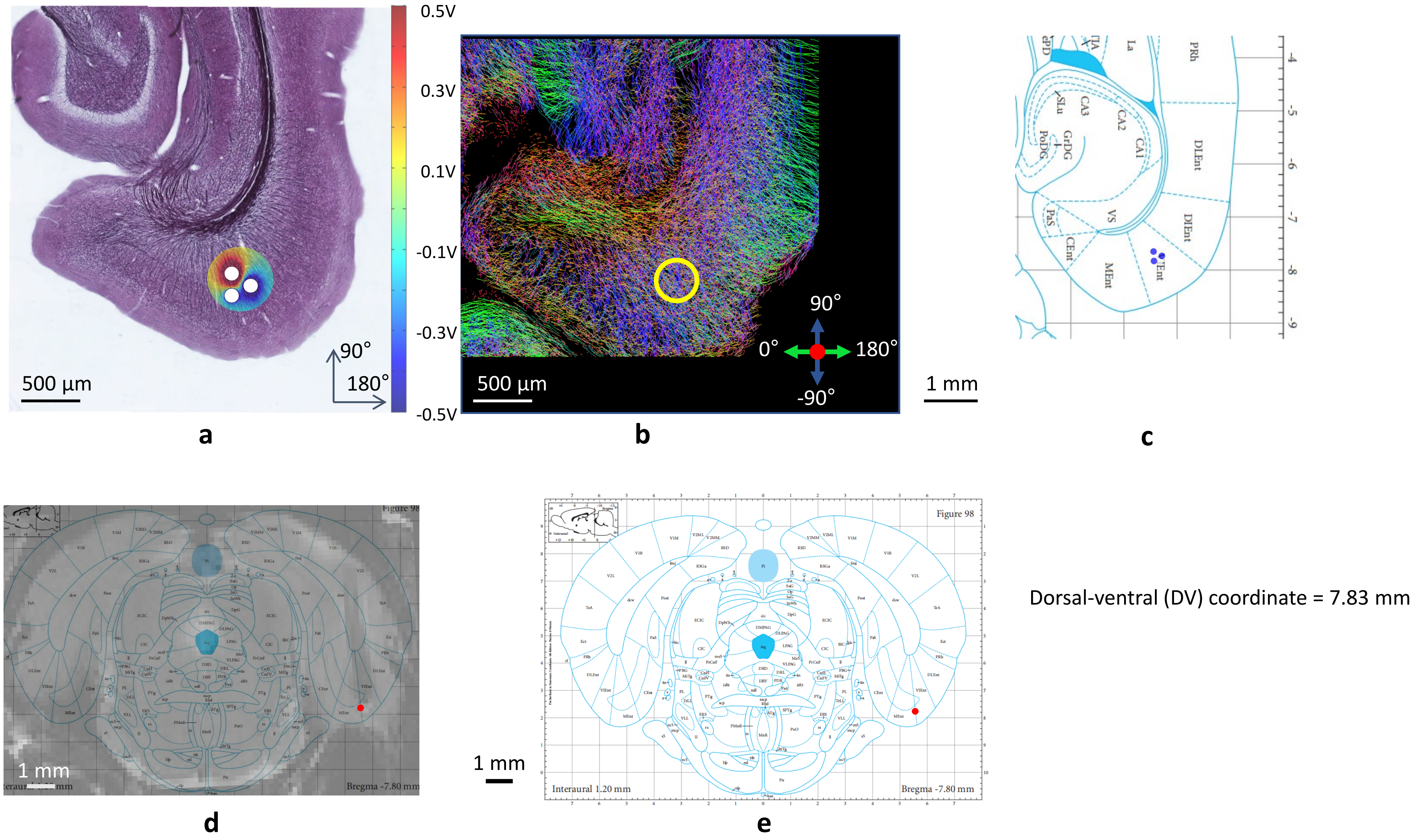

Dorsal-ventral (DV) coordinate = 7.83 mm

EC group

Rat 5, 1.8 mA

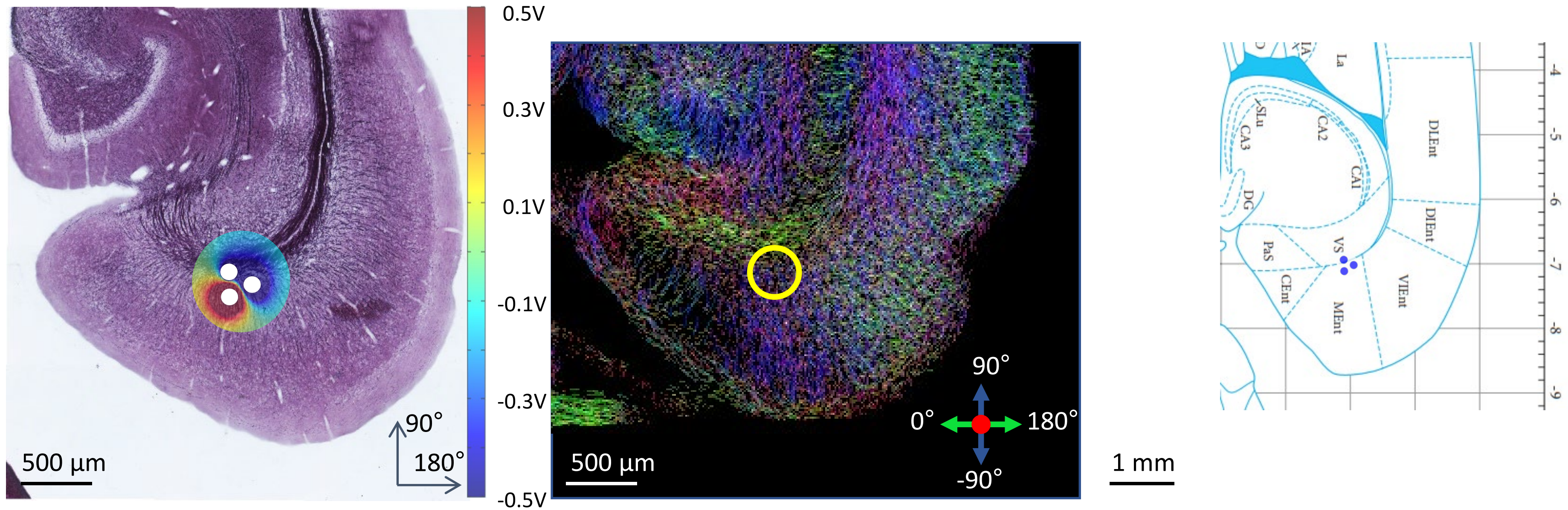

a

b

c

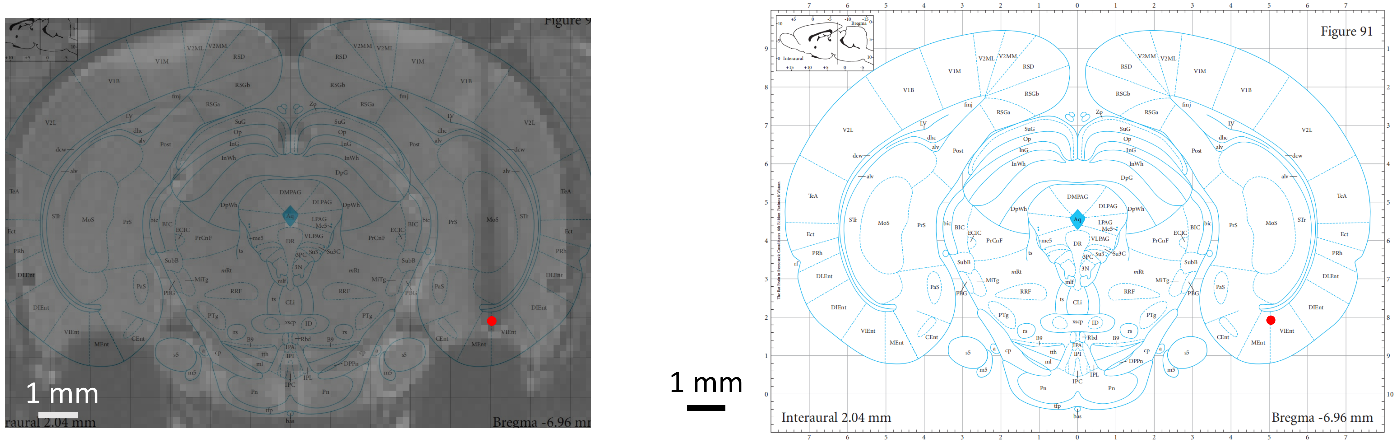

d

e

Dorsal-ventral (DV) coordinate = 8.1 mm

EC group

Rat 6, 1.2 mA

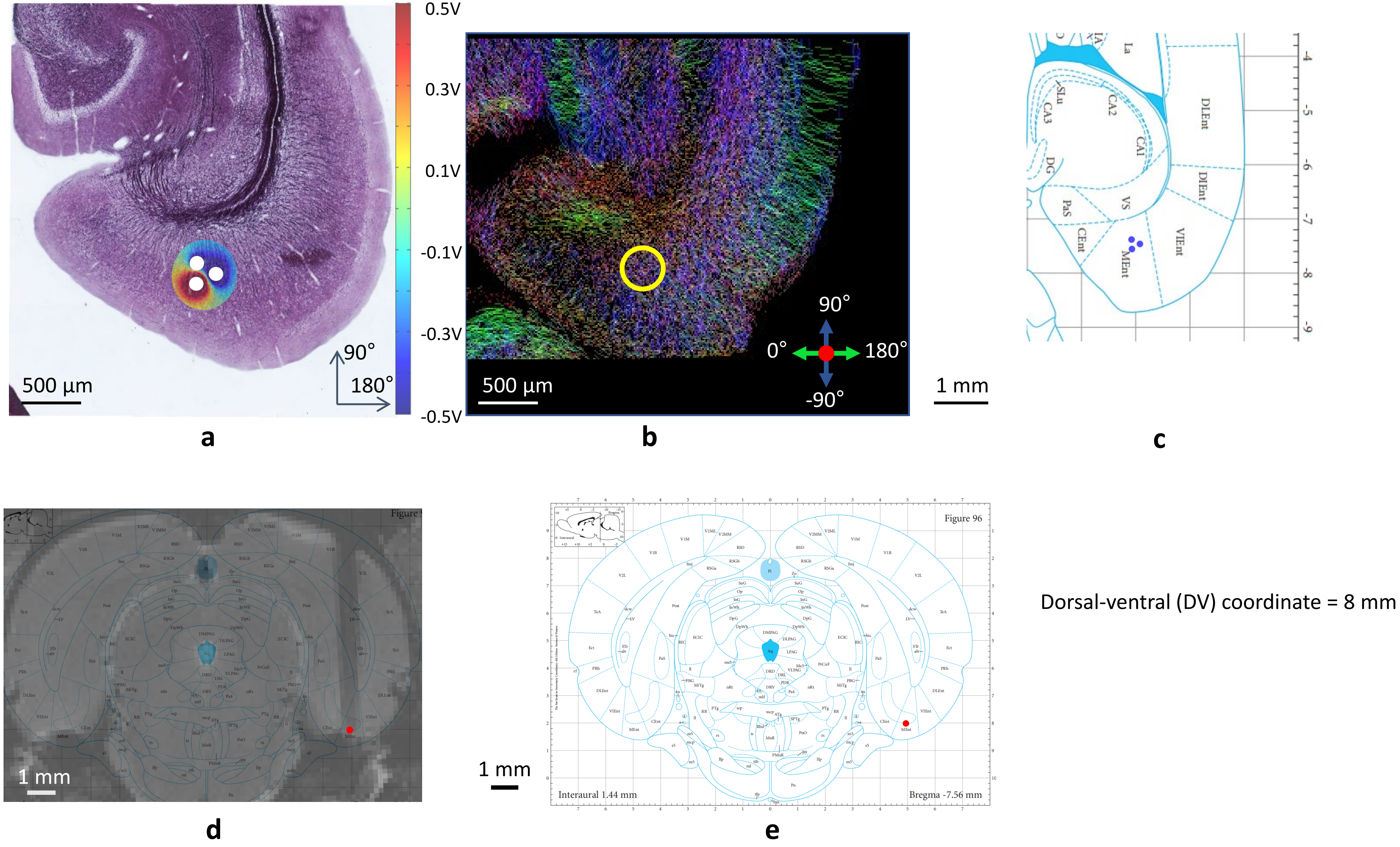

EC group

Rat 7, 2.0 mA

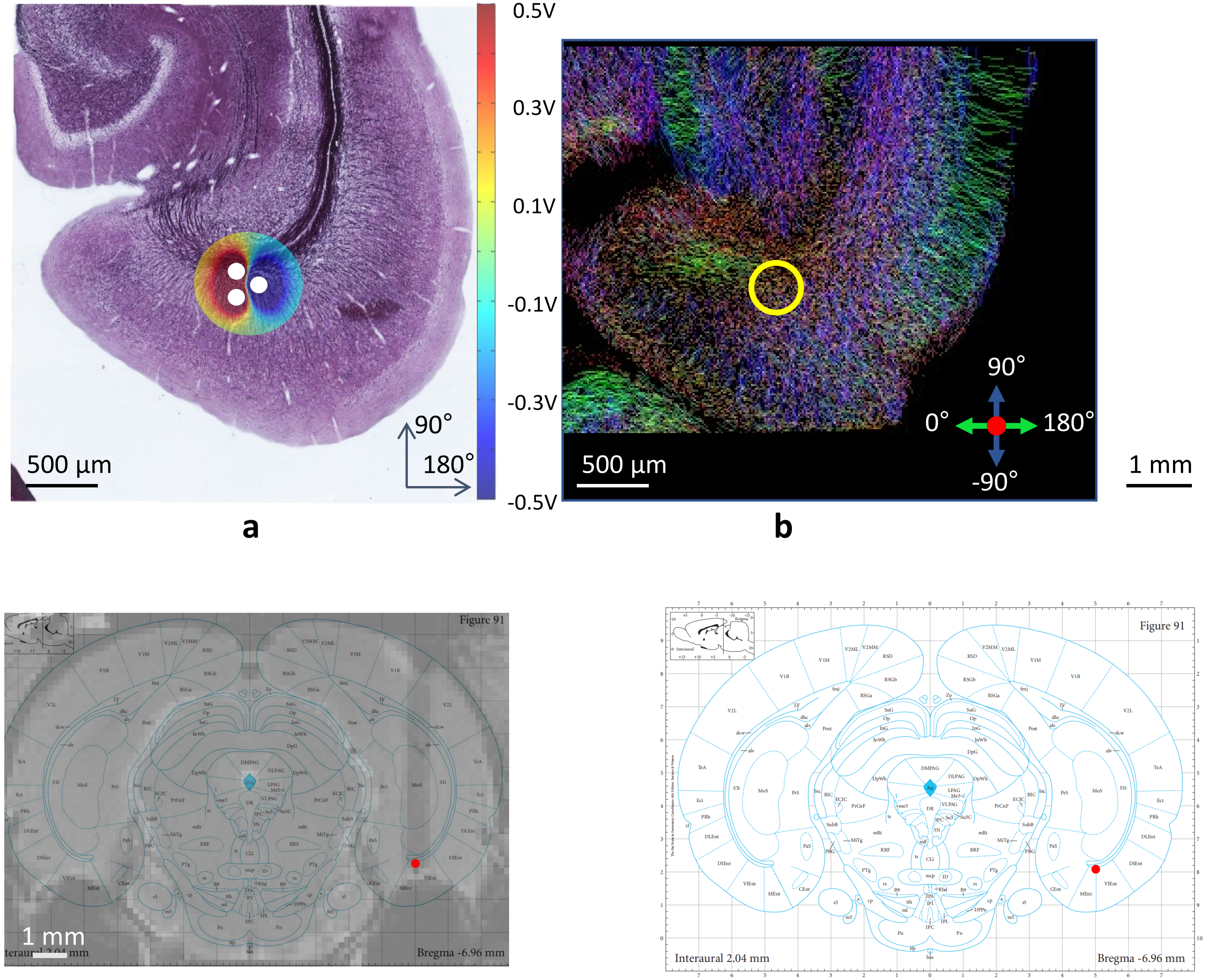

a b c d e

Dorsal-ventral (DV) coordinate = 8 mm

EC group

Rat 8, 2.0 mA

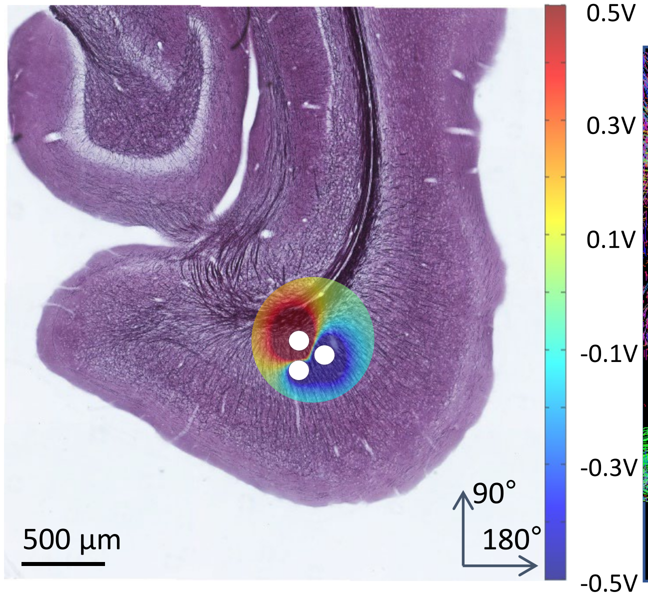

a

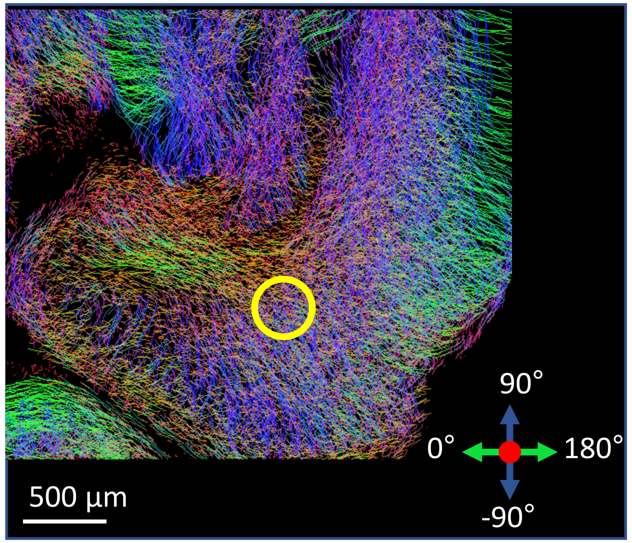

b

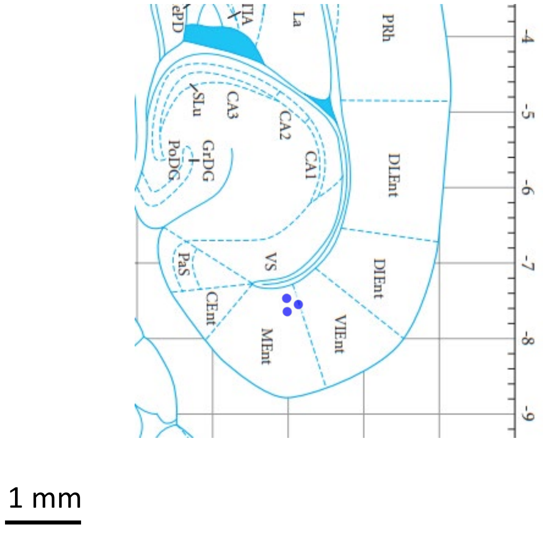

c

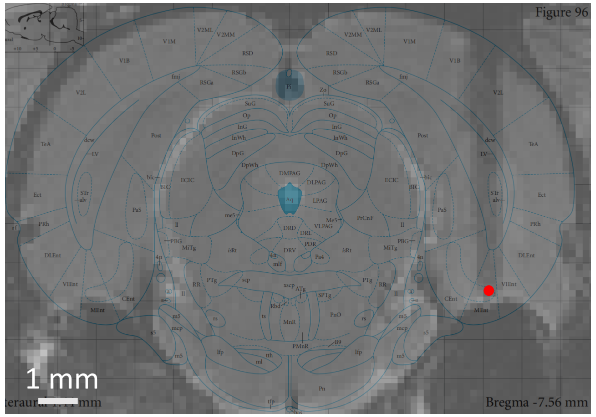

d

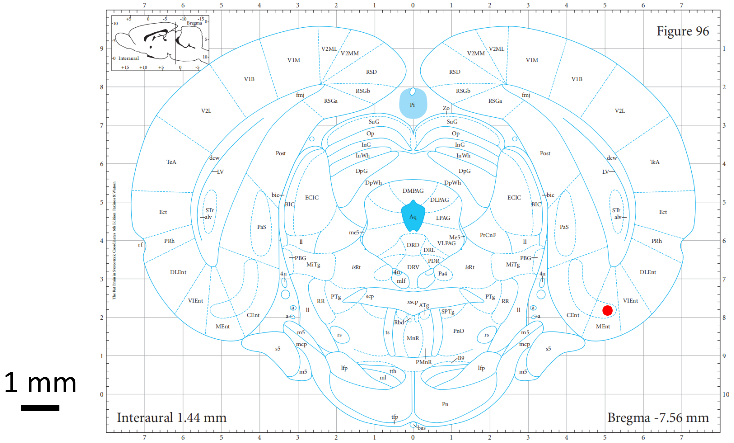

e

Dorsal-ventral (DV) coordinate = 7.8 mm

EC group

Rat 9, 2.0 mA

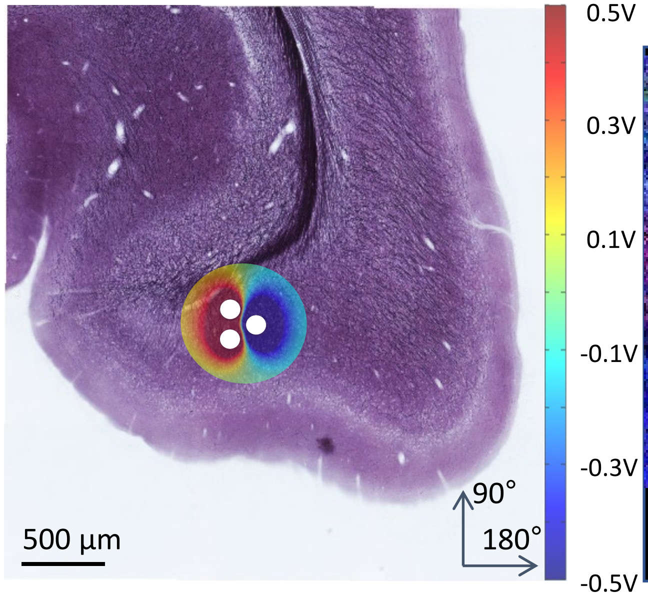

a

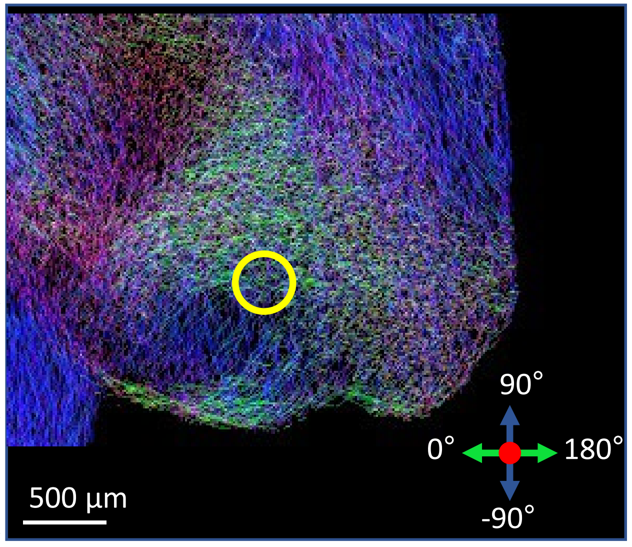

b

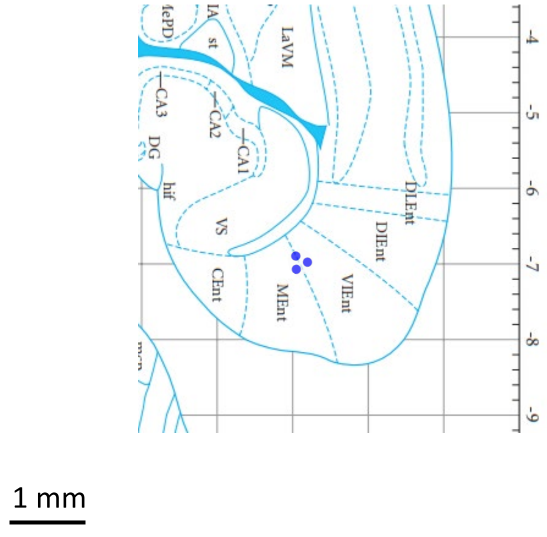

c

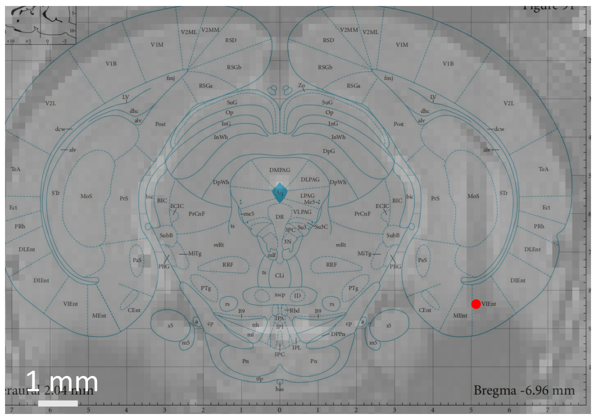

d

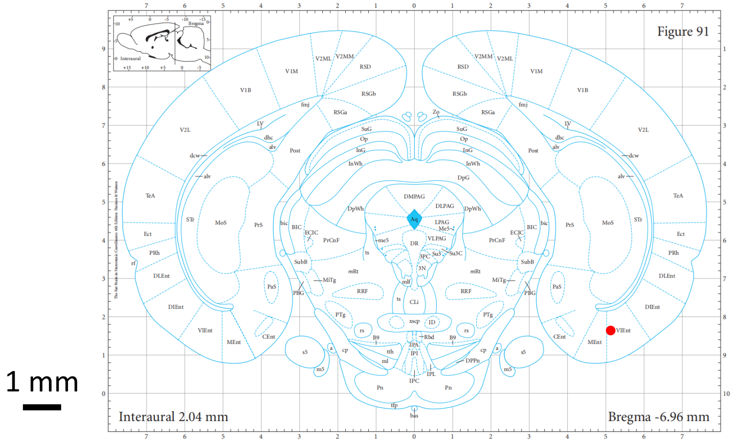

e

Dorsal-ventral (DV) coordinate = 8.4 mm

EC group

Rat 10, 2.0 mA

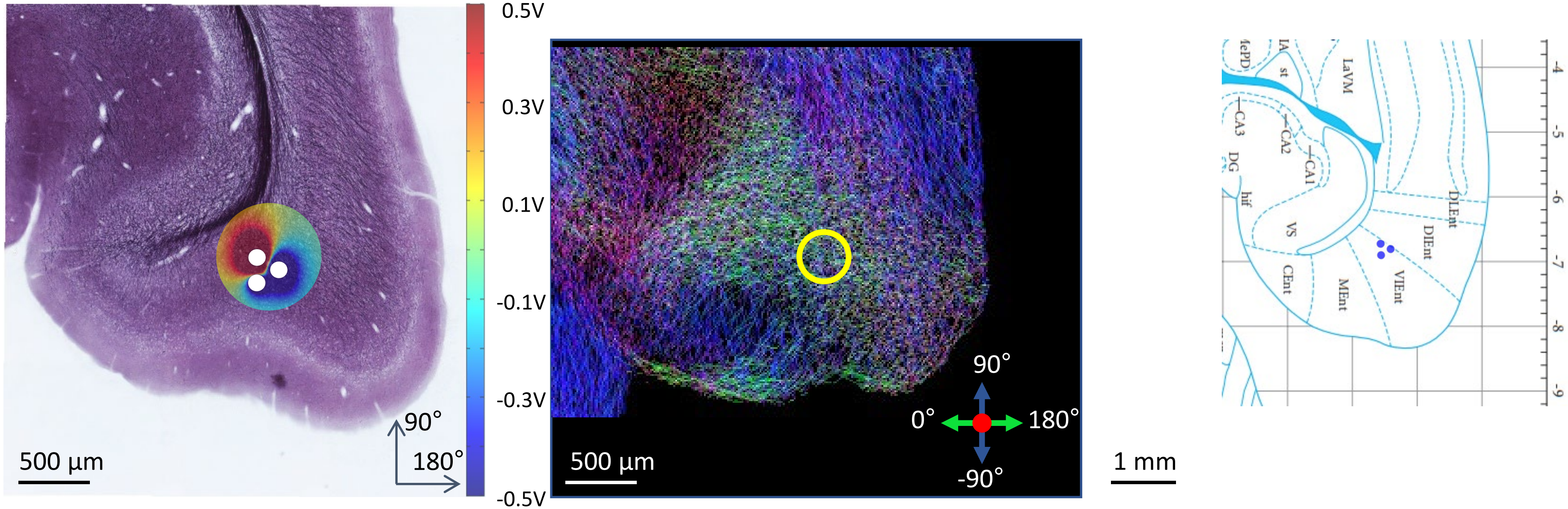

a b c

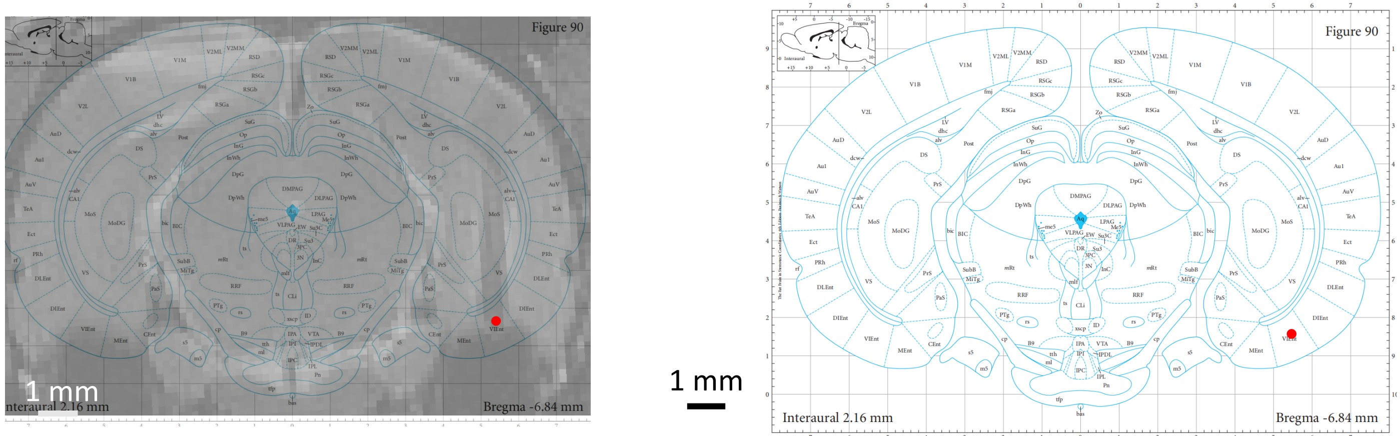

d e

Dorsal-ventral (DV) coordinate = 8.4 mm

Supplementary Figure 2: electrode location in MSN

a) Tractogram in horizontal view. The yellow circle shows the size and location of 3-electrode bundle.
b) Estimated location of the electrode on an horizontal view from the atlas by Paxinos and Watson (2007) “The Rat Brain in Stereotaxic Coordinates (6th Edition)”. Blue circles indicate the size and location of the 3-channel bundle.
c) Tractogram in coronal view. The yellow point shows the center of the 3-electrode bundle.
d) MRI image in coronal view overlaid on the atlas by Paxinos and Watson (2007) “The Rat Brain in Stereotaxic Coordinates (6th Edition)”. The red point indicates the tip of the electrode. The MRI image is displayed in neurological convention (left side of the image corresponds to the left side of the brain).
e) Estimated location of the electrode on the same coronal view of the atlas by Paxinos and Watson (2007) “The Rat Brain in Stereotaxic Coordinates (6th Edition)” as in d). The red point indicates the tip of electrode.

The rat's number and the used current amplitude are indicated on the top left. The dorsal-ventral (DV) coordinate of the electrode tip is indicated on the bottom, right. Atlas images by Paxinos and Watson (2007) “The Rat Brain in Stereotaxic Coordinates (6th Edition)” are taken with permission.

MSN group

Rat 11, 0.6 mA

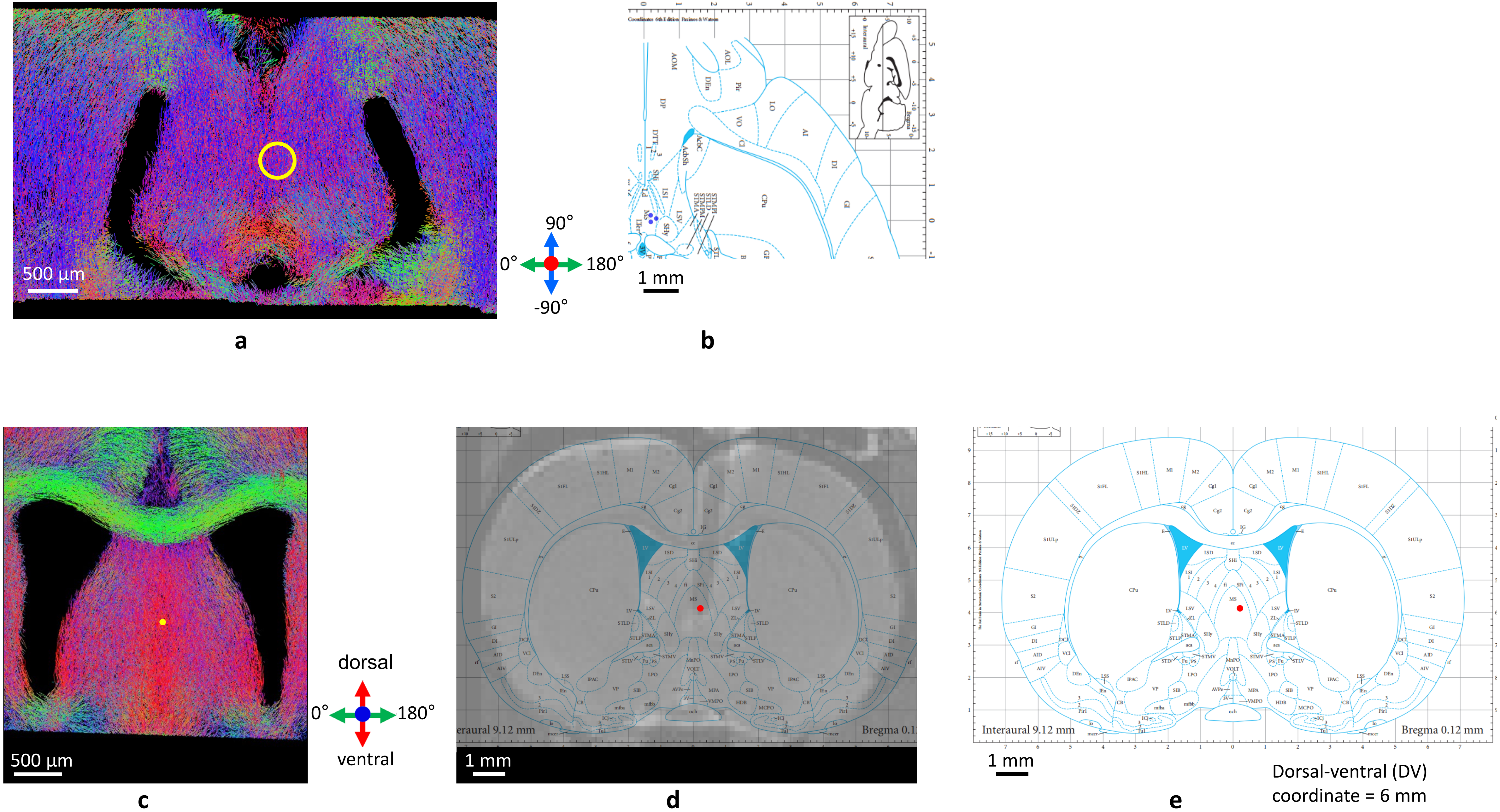

MSN group

Rat 12, 1.1 mA

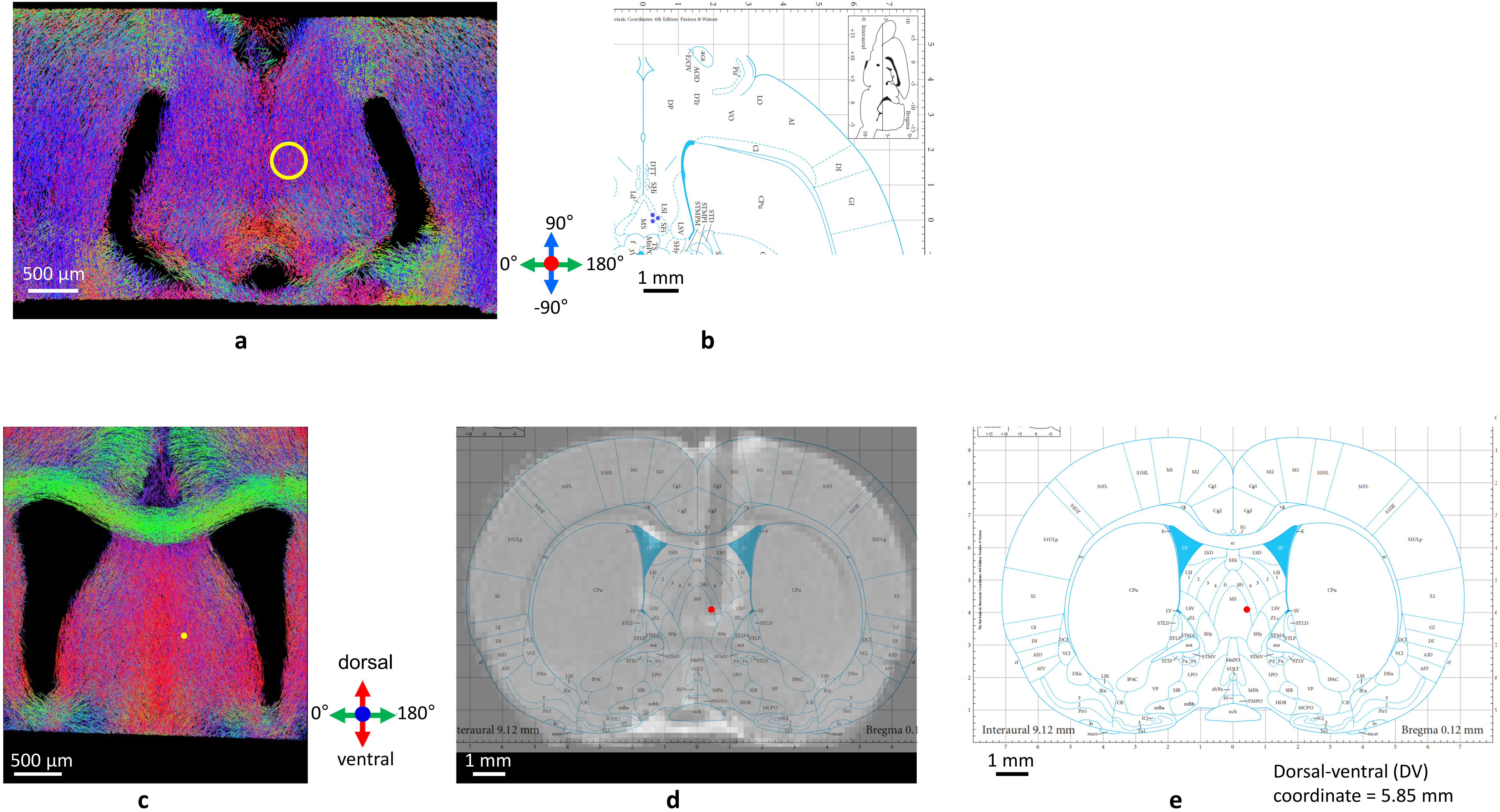

MSN group

Rat 13, 0.7 mA

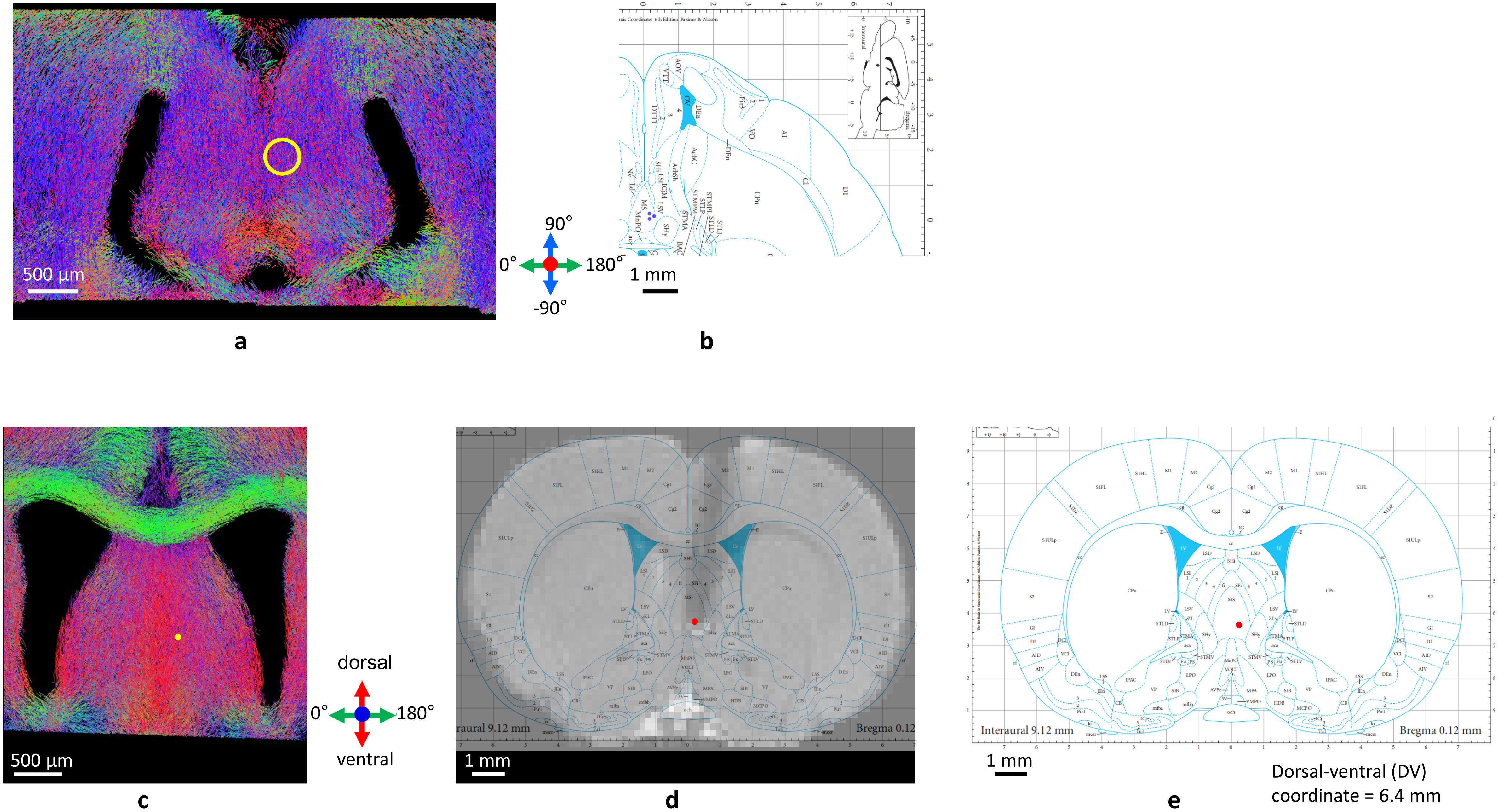

Dorsal-ventral (DV) coordinate = 6.4 mm

MSN group

Rat 14, 0.8 mA

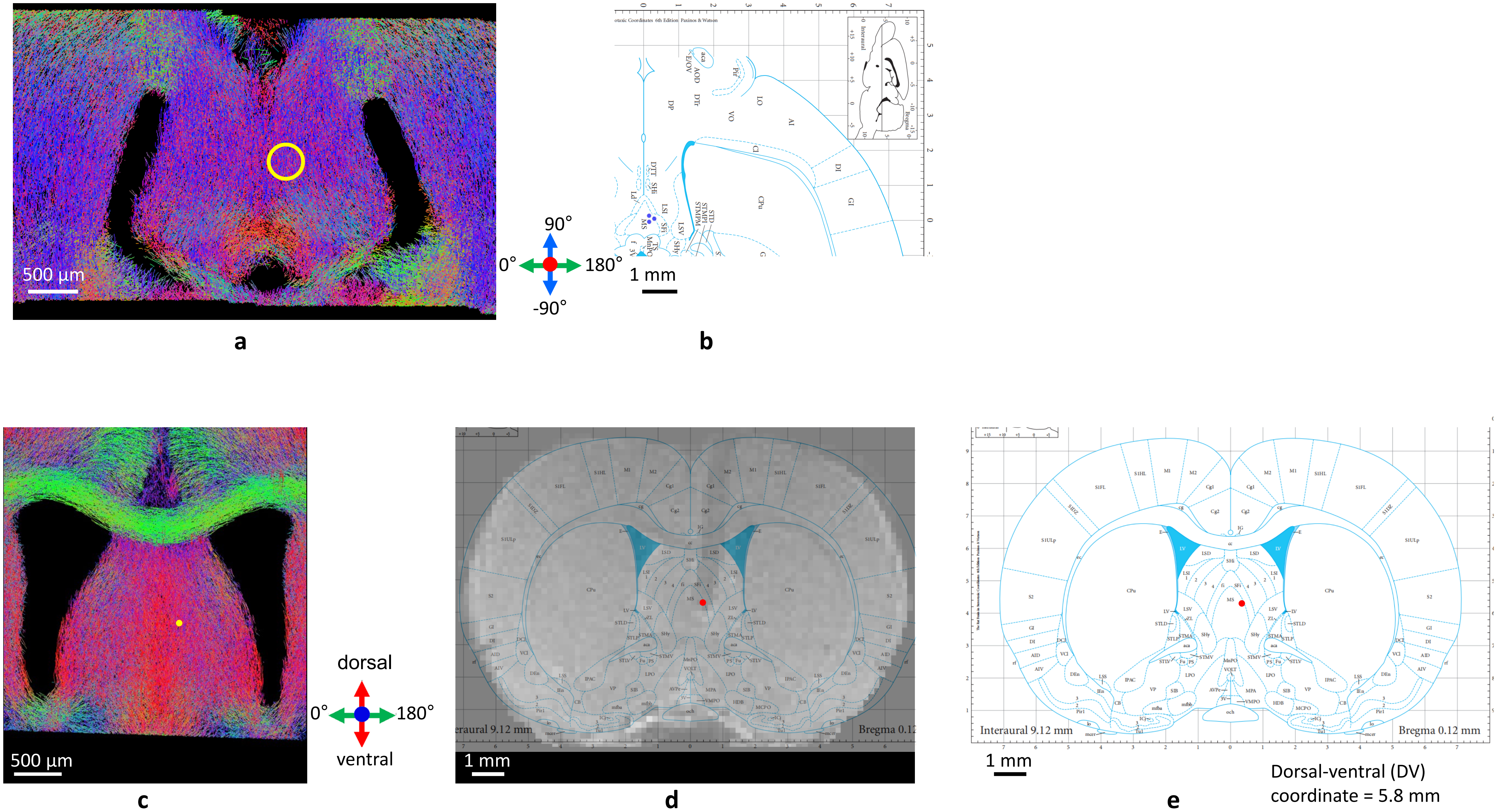

MSN group

Rat 15, 1.3 mA

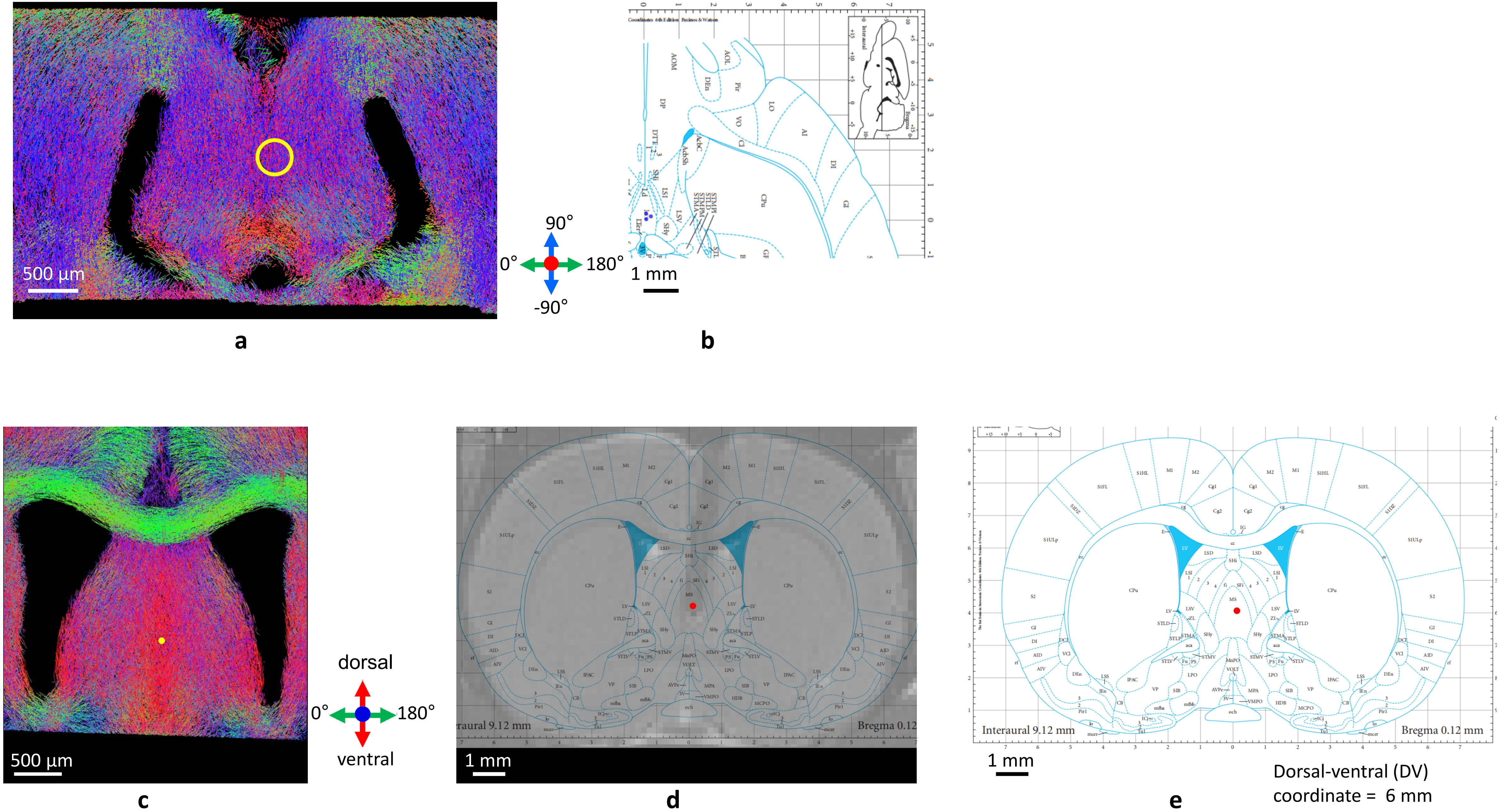

MSN group

Rat 16, 1.15 mA

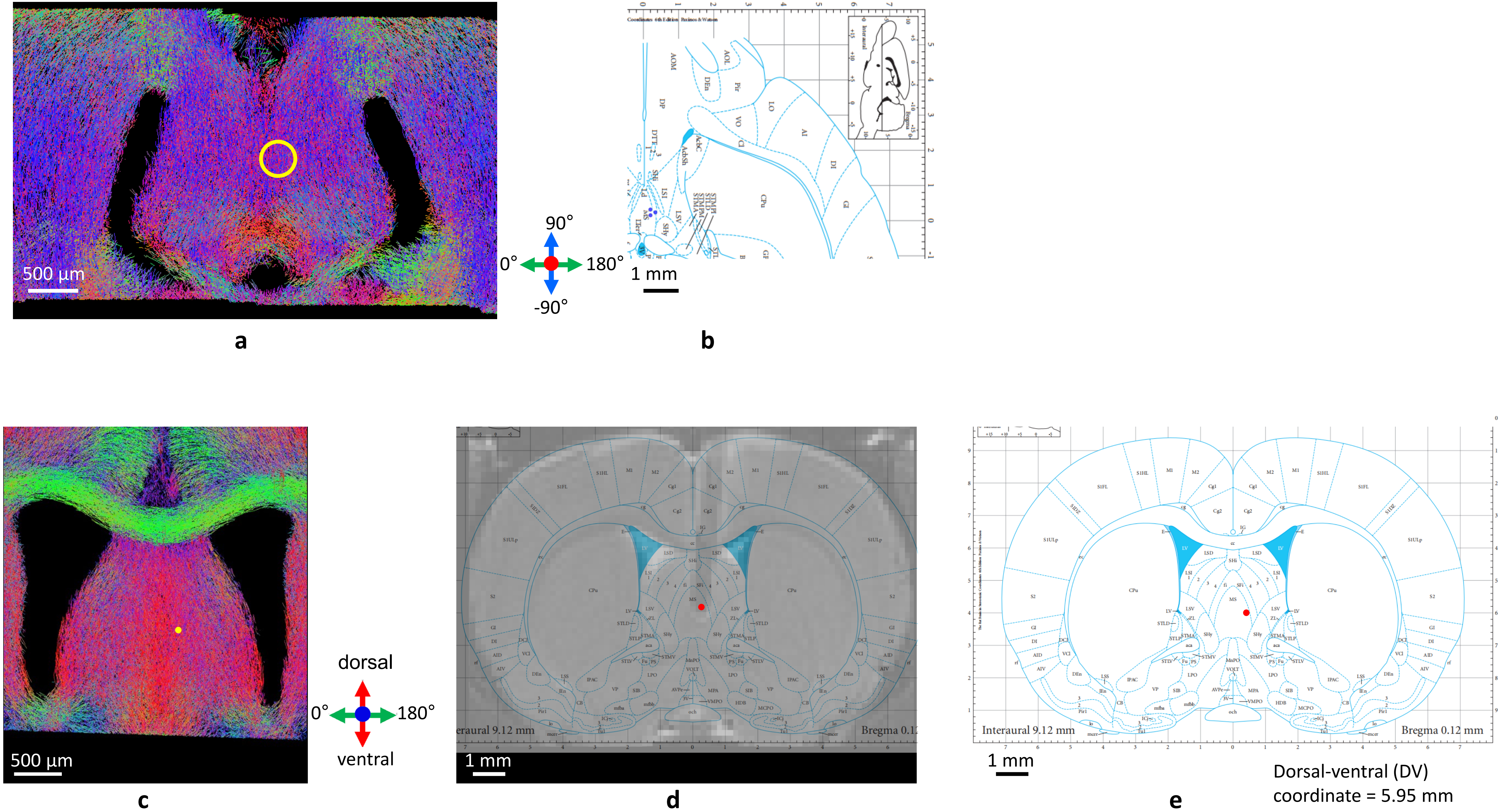

MSN group

Rat 17, 1.0 mA

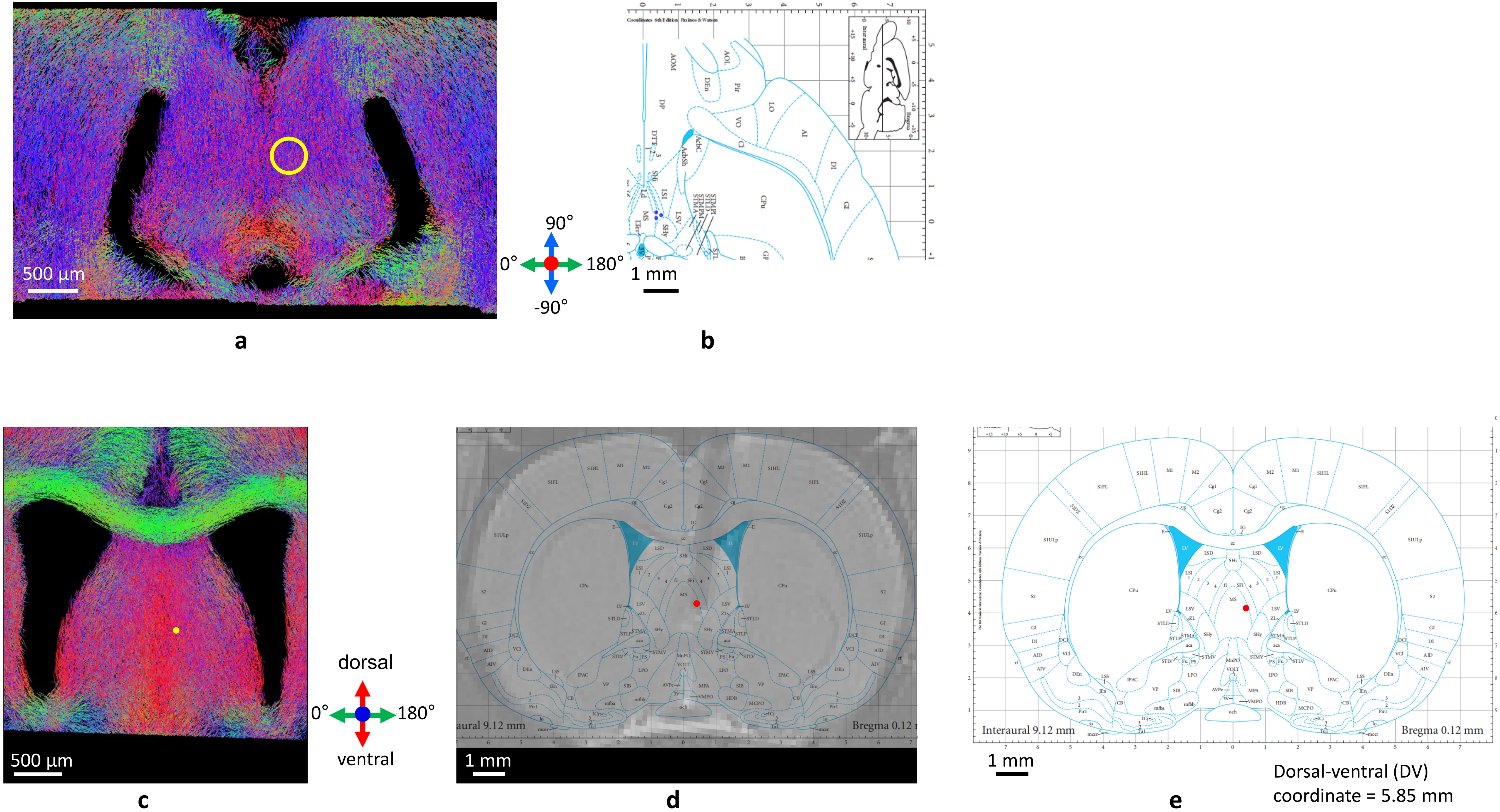

MSN group

Rat 18, 1.25 mA

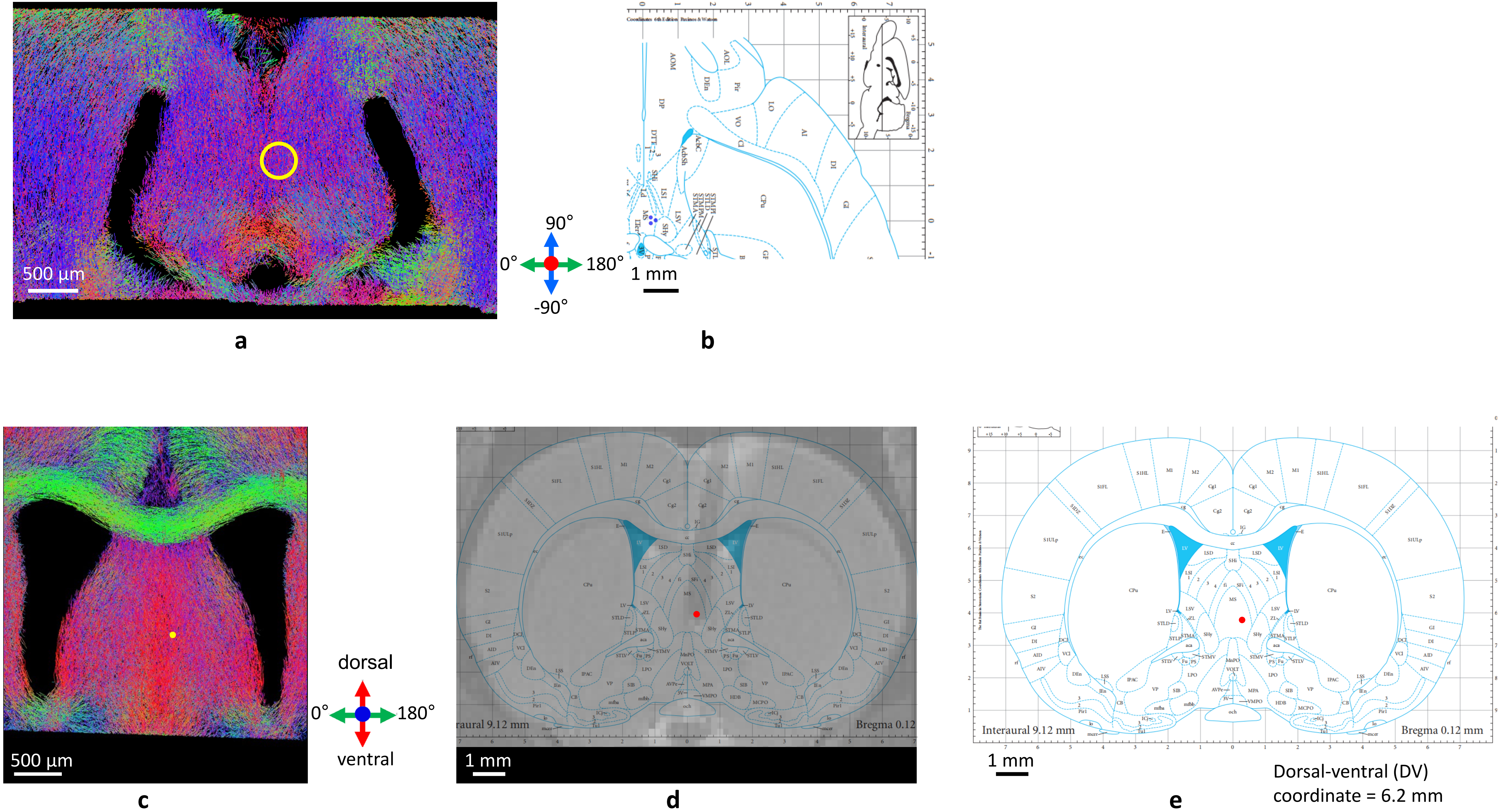

Supplementary Figure 3: Individual fMRI responses during OS-DBS of the right EC

Group, rat number, and current amplitude are indicated on the top, while acquisition order is indicated on the bottom. fMRI maps are thresholded at $p \leq 0.05$, FWE corrected (processing details are available in the main text). Stimulation angles and field distributions on an axial plane are indicated on the top. Brain images are displayed in neurological convention (left side of the image corresponds to the left side of the brain). No brain mask was applied.

EC group

Rat 1, 1.5 mA

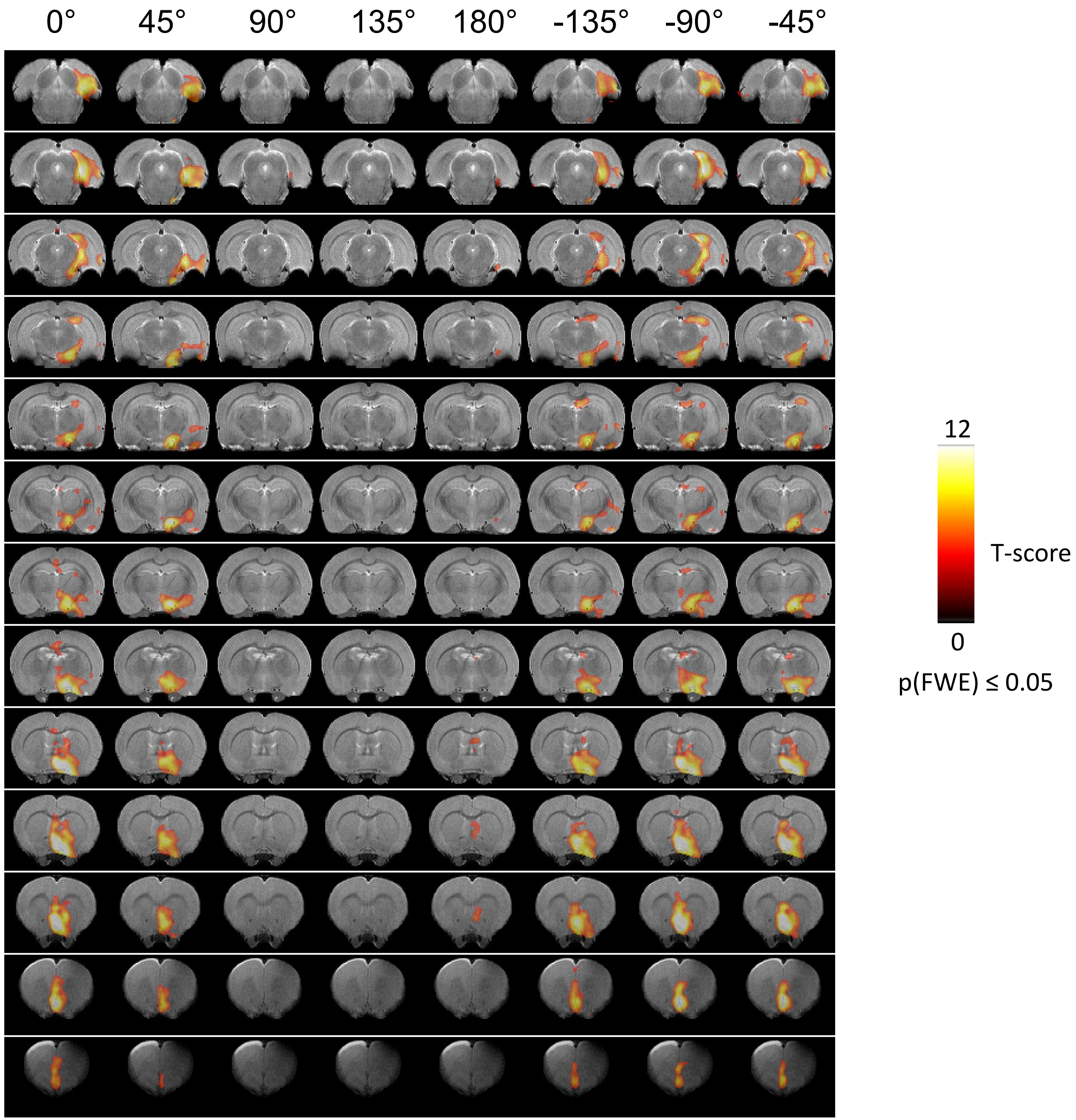

**Order**	**1**	**2**	**3**	**4**	**5**	**6**	**7**	**8**
Angle(°)	-90	135	90	0	-45	-135	180	45

EC group
Rat 2, 1.0 mA

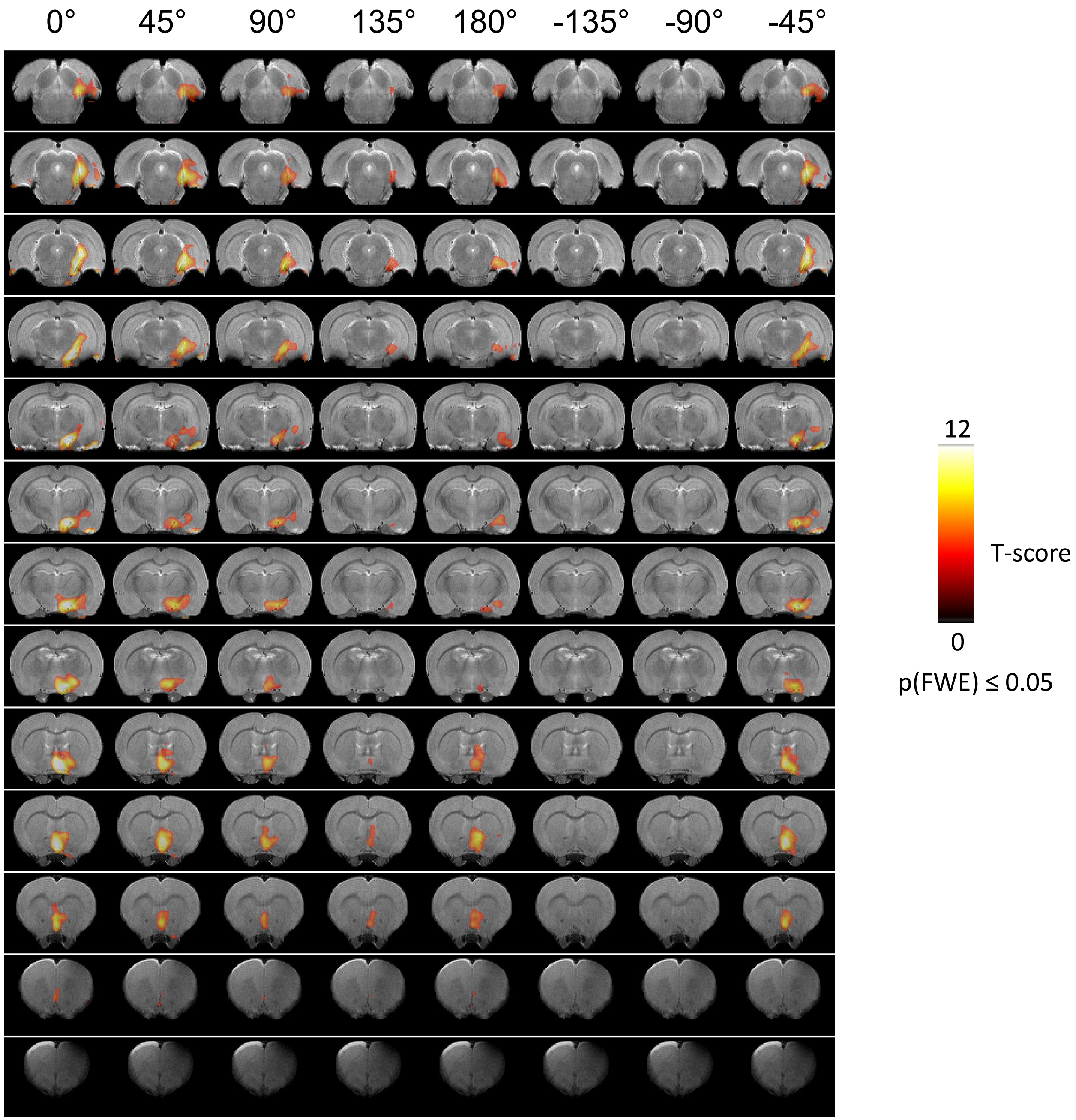

**Order**	**1**	**2**	**3**	**4**	**5**	**6**	**7**	**8**
Angle(°)	0	90	-135	-90	45	180	135	-45

EC group

Rat 3, 1.25 mA

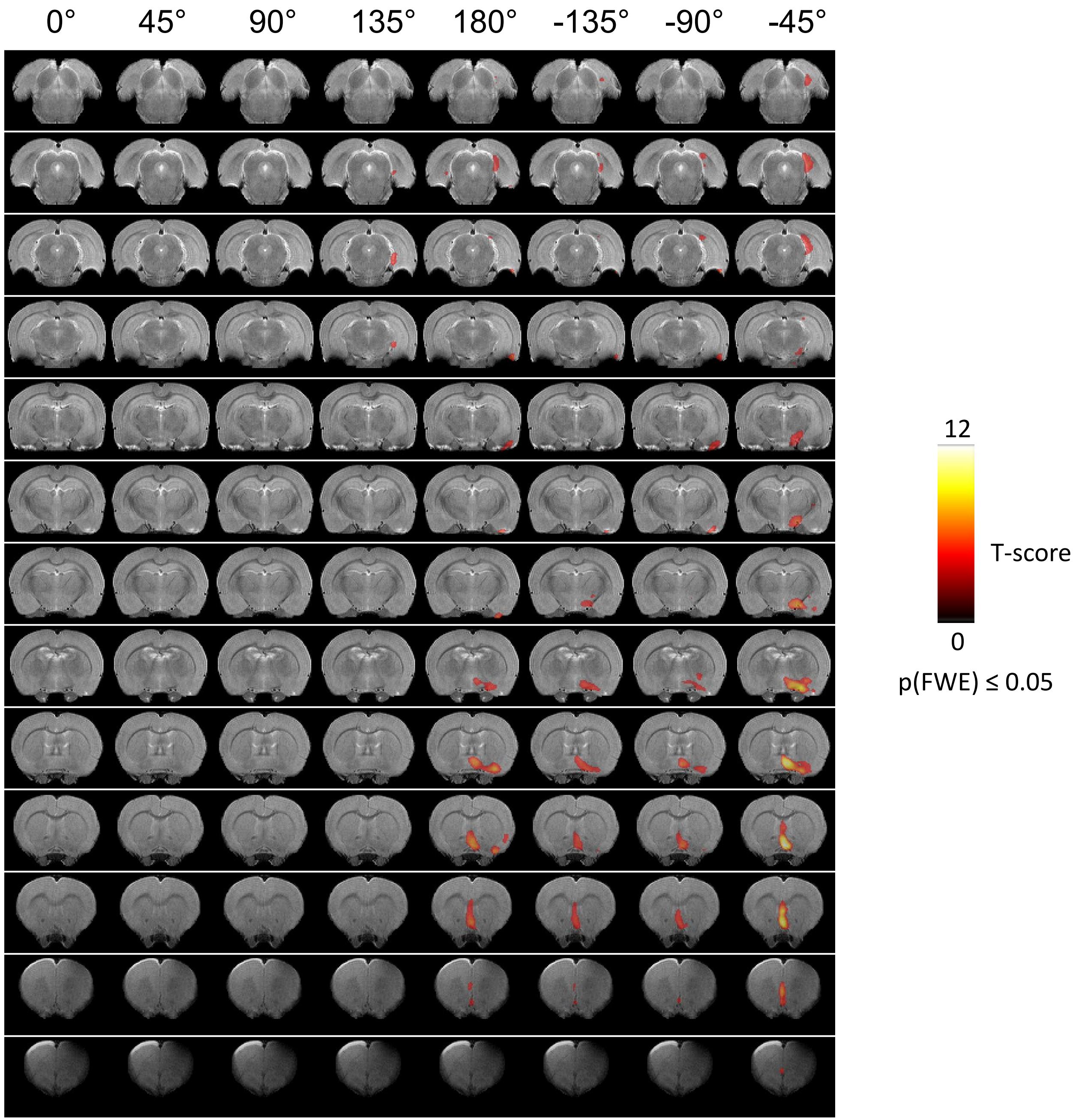

Order	1	2	3	4	5	6	7	8
Angle(°)	180	-45	135	-90	-135	90	0	45

EC group

Rat 4, 1.75 mA

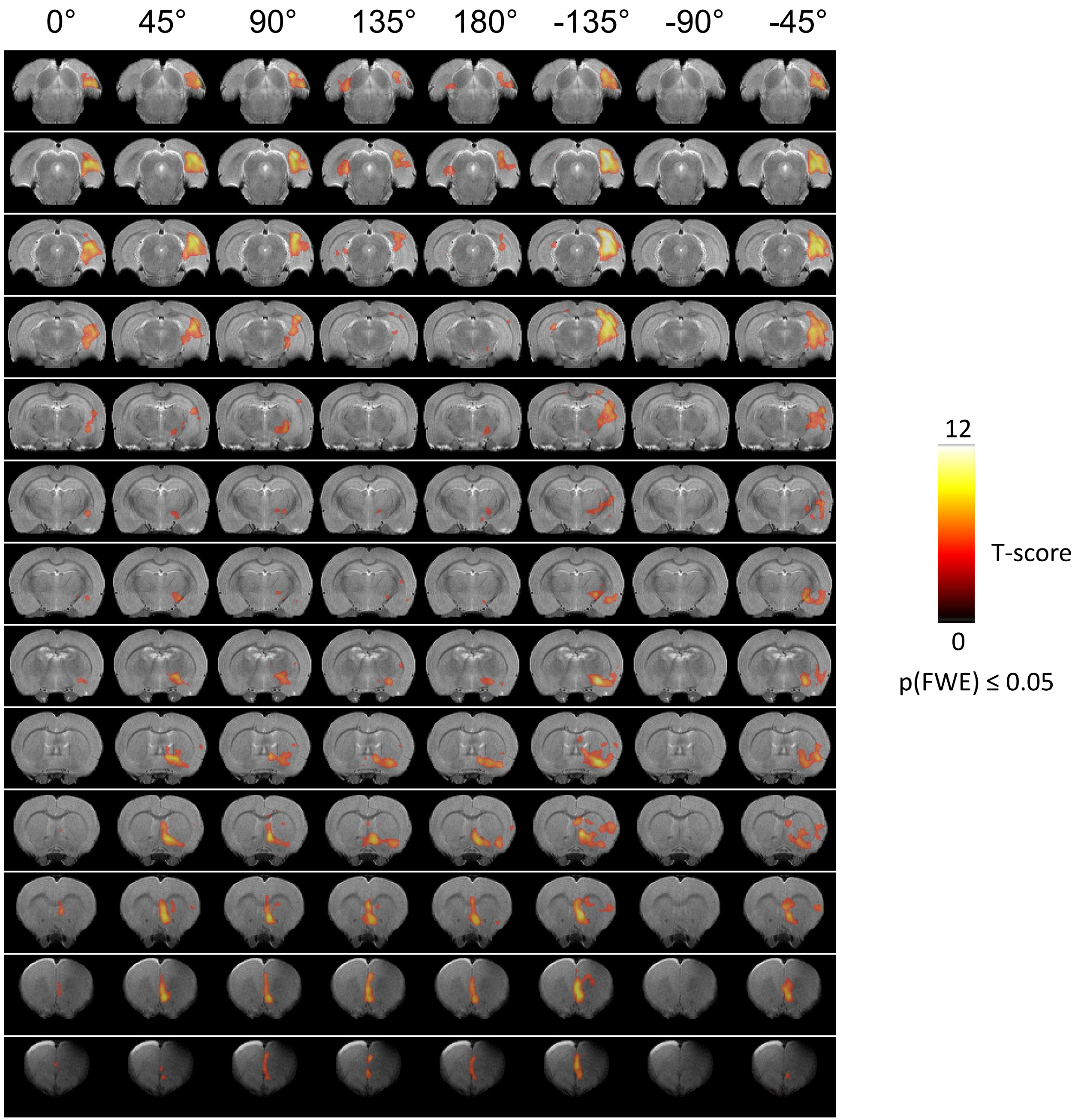

**Order**	**1**	**2**	**3**	**4**	**5**	**6**	**7**	**8**
Angle(°)	180	-45	45	-90	0	135	90	-135

EC group

Rat 5, 1.8 mA

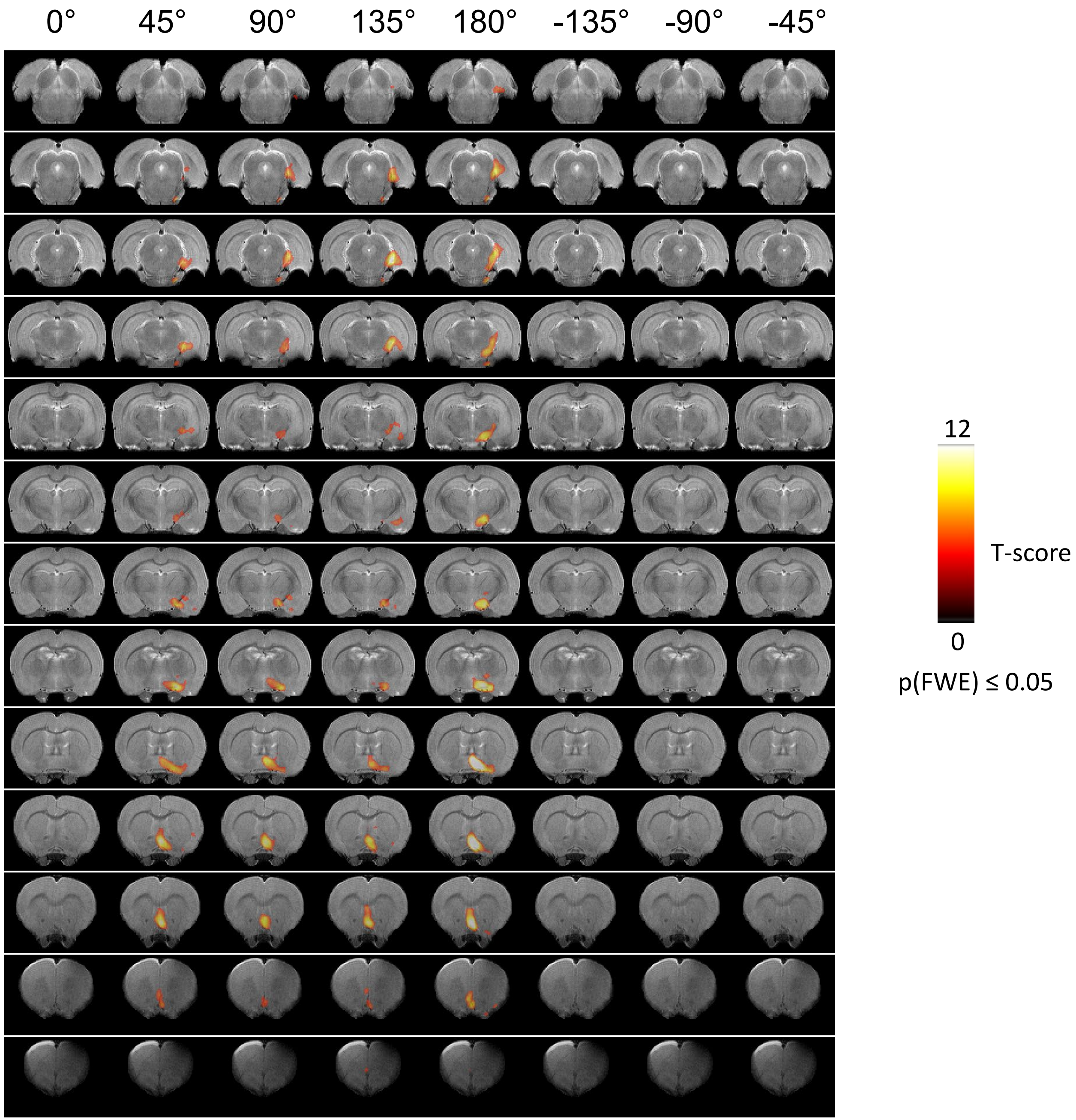

**Order**	**1**	**2**	**3**	**4**	**5**	**6**	**7**	**8**
Angle(°)	180	-90	0	45	-45	135	-135	90

EC group

Rat 6, 1.2 mA

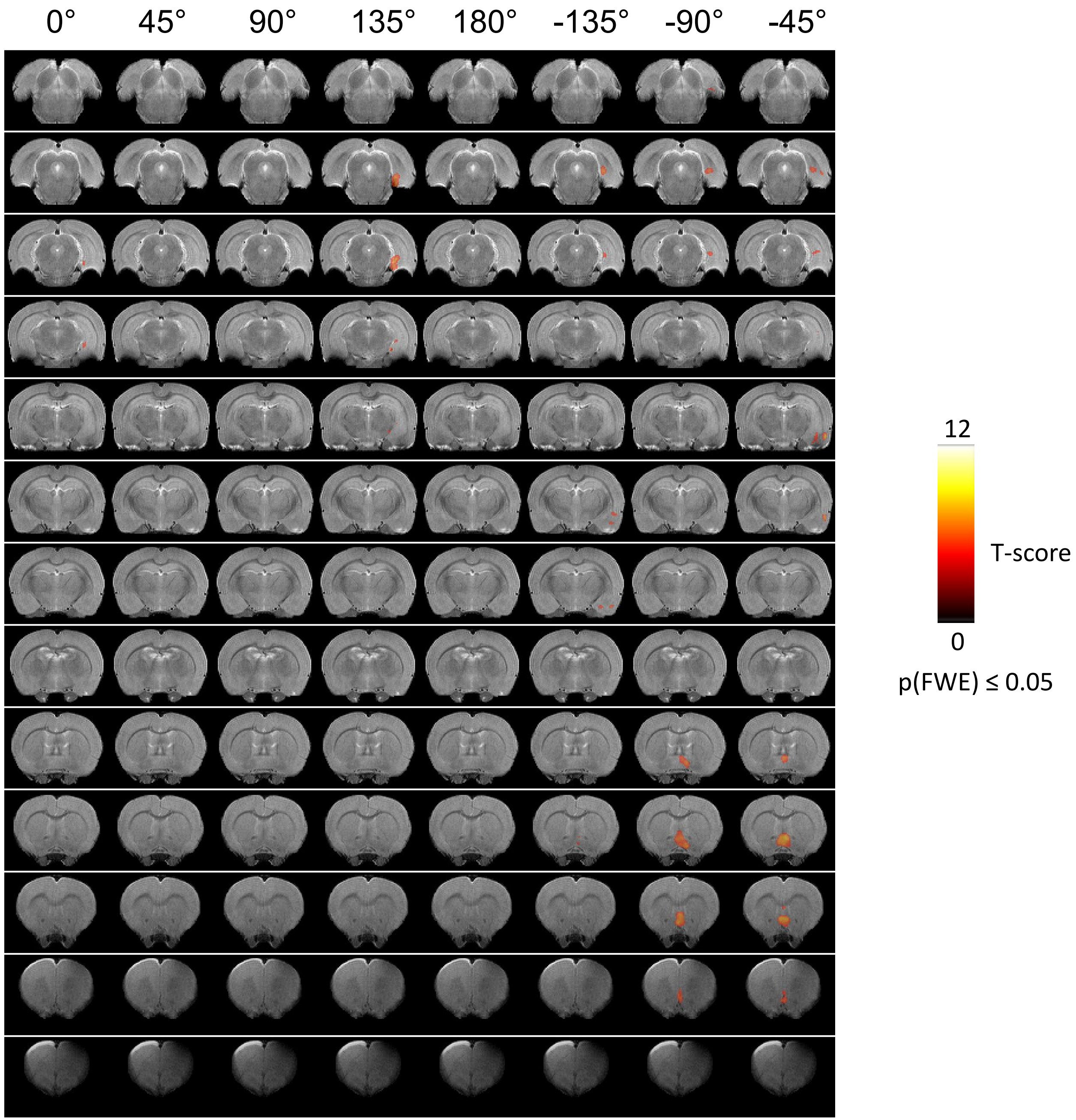

Order	1	2	3	4	5	6	7	8
Angle(°)	180	45	-135	90	-90	-45	0	135

EC group
Rat 7, 2.0 mA

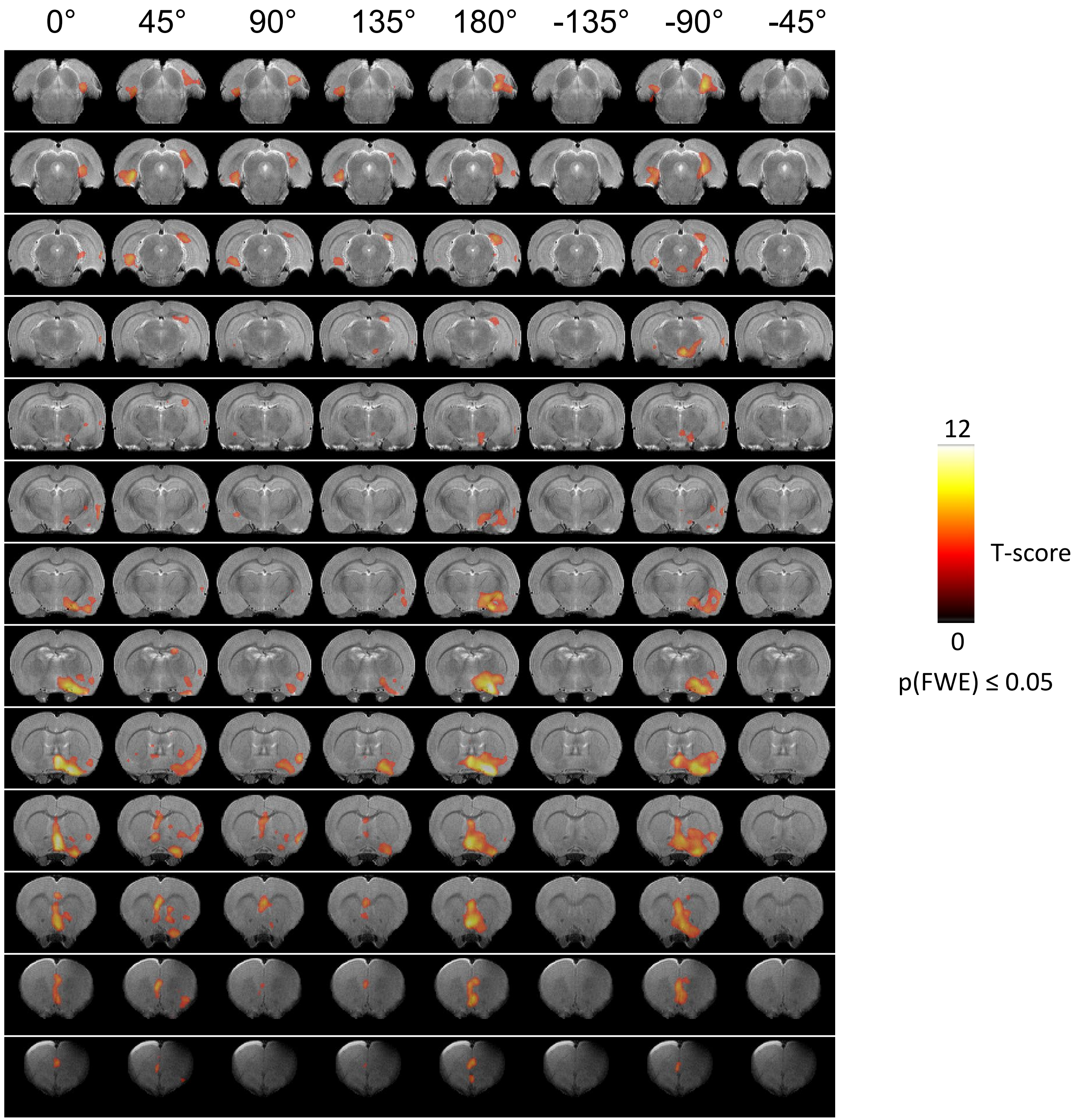

Order	1	2	3	4	5	6	7	8
Angle(°)	180	135	0	-90	45	-135	-45	90

EC group

Rat 8, 2.0 mA

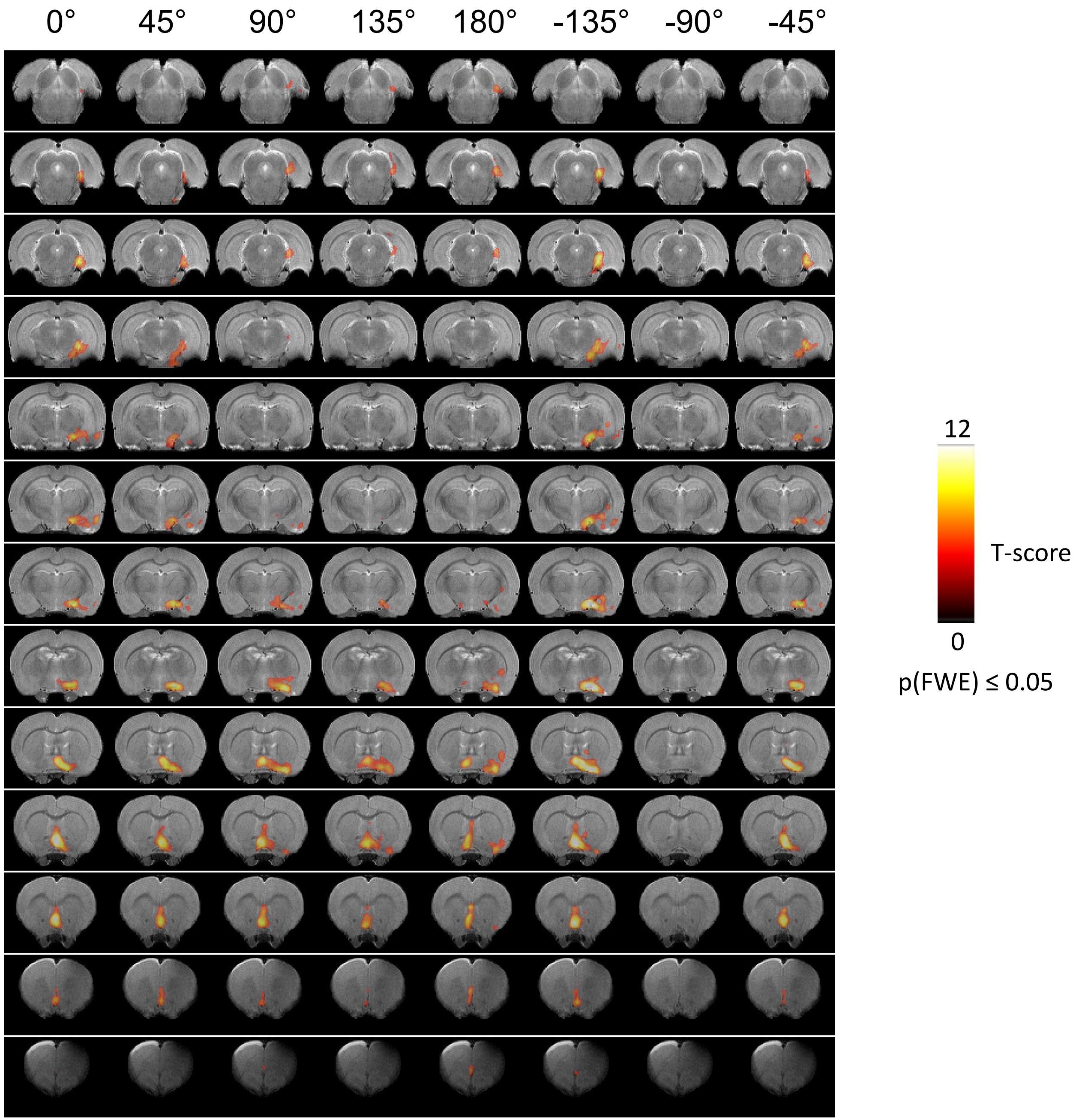

**Order**	**1**	**2**	**3**	**4**	**5**	**6**	**7**	**8**
Angle(°)	180	-90	0	45	-135	-45	90	135

EC group

Rat 9, 2.0 mA

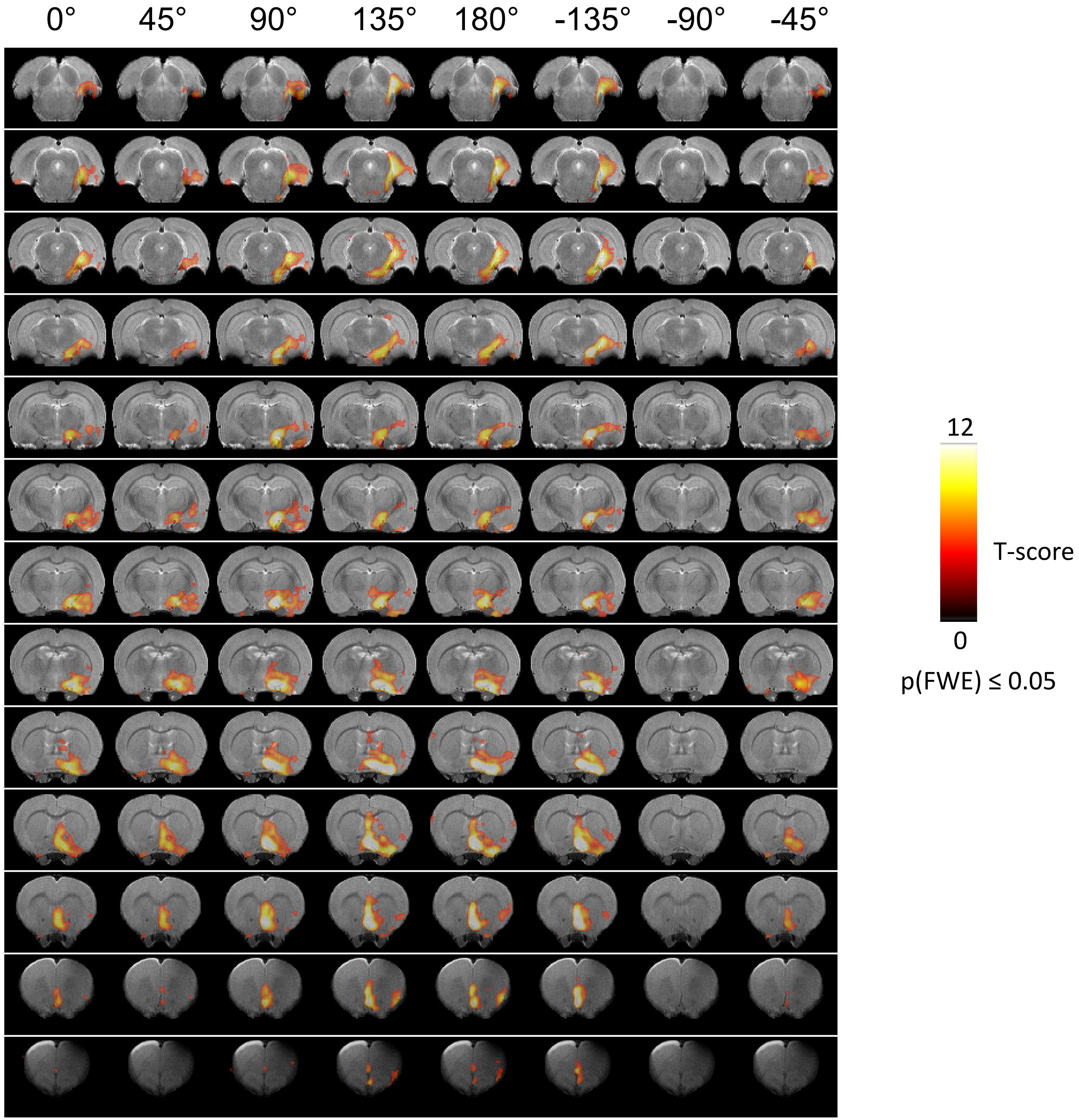

**Order**	**1**	**2**	**3**	**4**	**5**	**6**	**7**	**8**
Angle(°)	180	0	-135	90	135	45	-45	-90

EC group

Rat 10, 2.0 mA

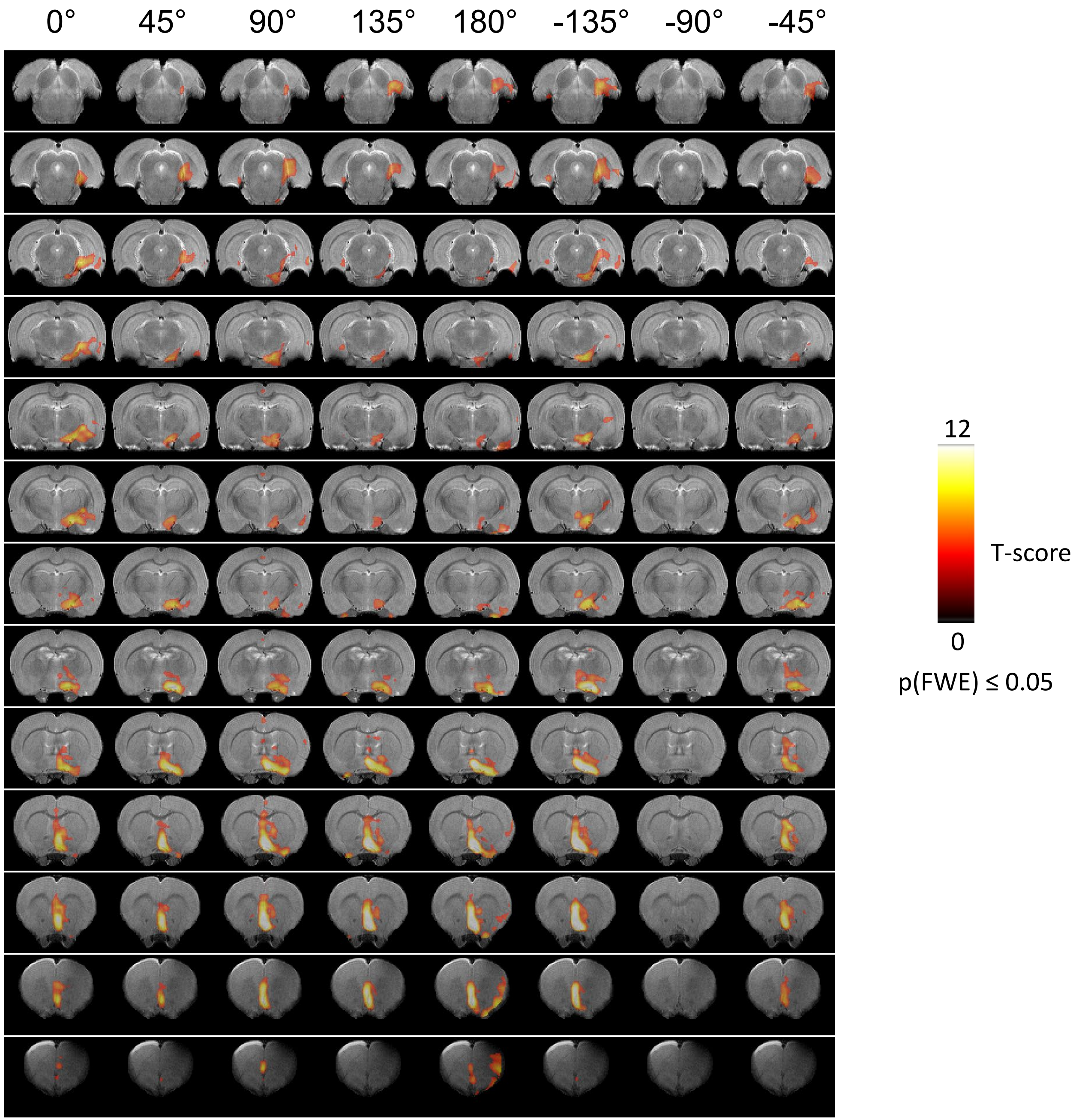

Order	1	2	3	4	5	6	7	8
Angle(°)	180	45	-90	0	135	-45	90	-135

Supplementary Figure 4: Individual fMRI responses during OS-DBS of MSN

Group, rat number, and current amplitude are indicated on the top, while acquisition order is indicated on the bottom. fMRI maps are thresholded at $p \leq 0.05$, FWE corrected (processing details are available in the main text). Stimulation angles and field distributions on an axial plane are indicated on the top. Brain images are displayed in neurological convention (left side of the image corresponds to the left side of the brain). No brain mask was applied.

MSN group
Rat 11, 0.6 mA

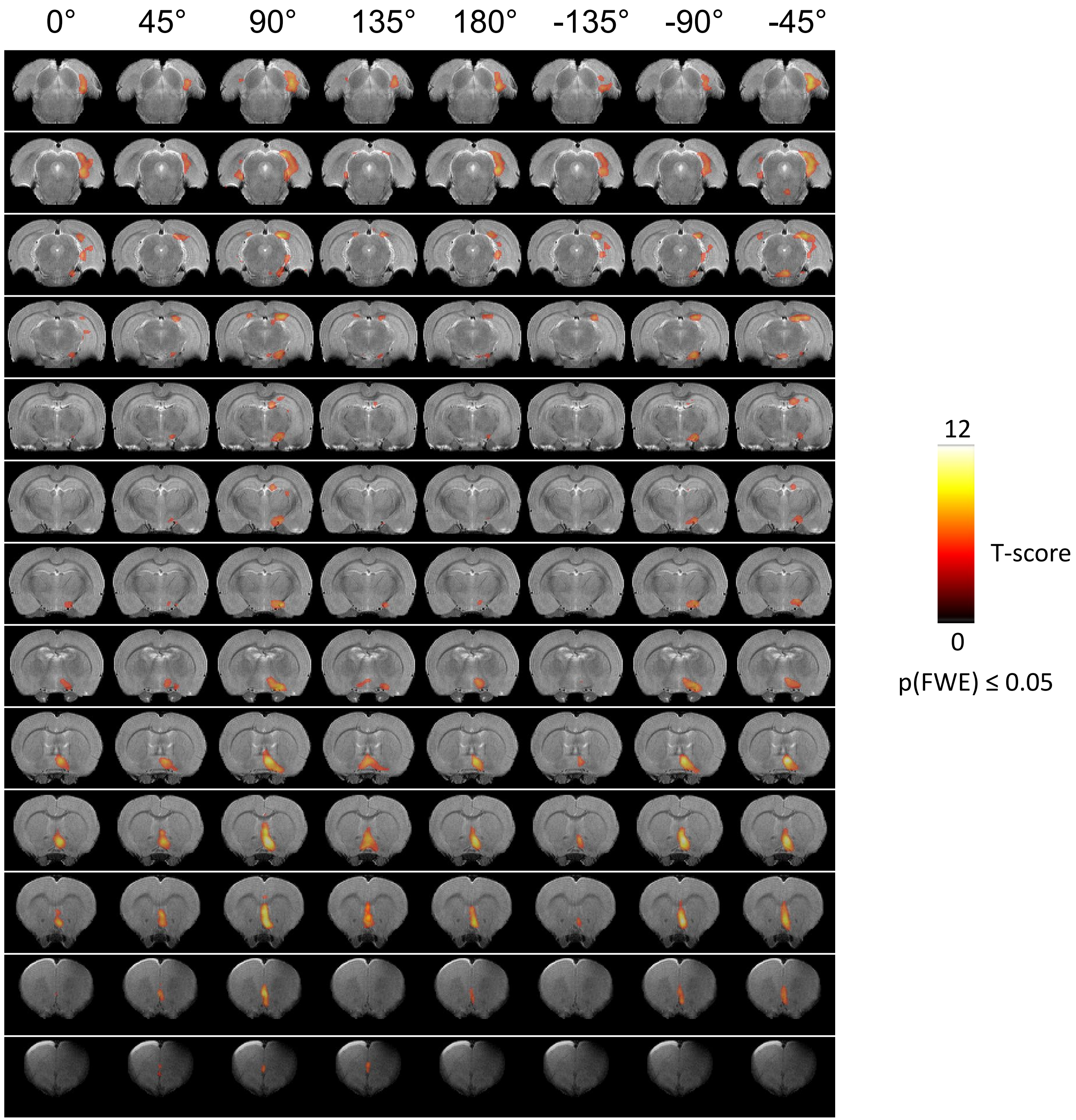

Order	1	2	3	4	5	6	7	8
Angle(°)	180	-90	90	-45	0	45	135	-135

MSN group

Rat 12, 1.1 mA

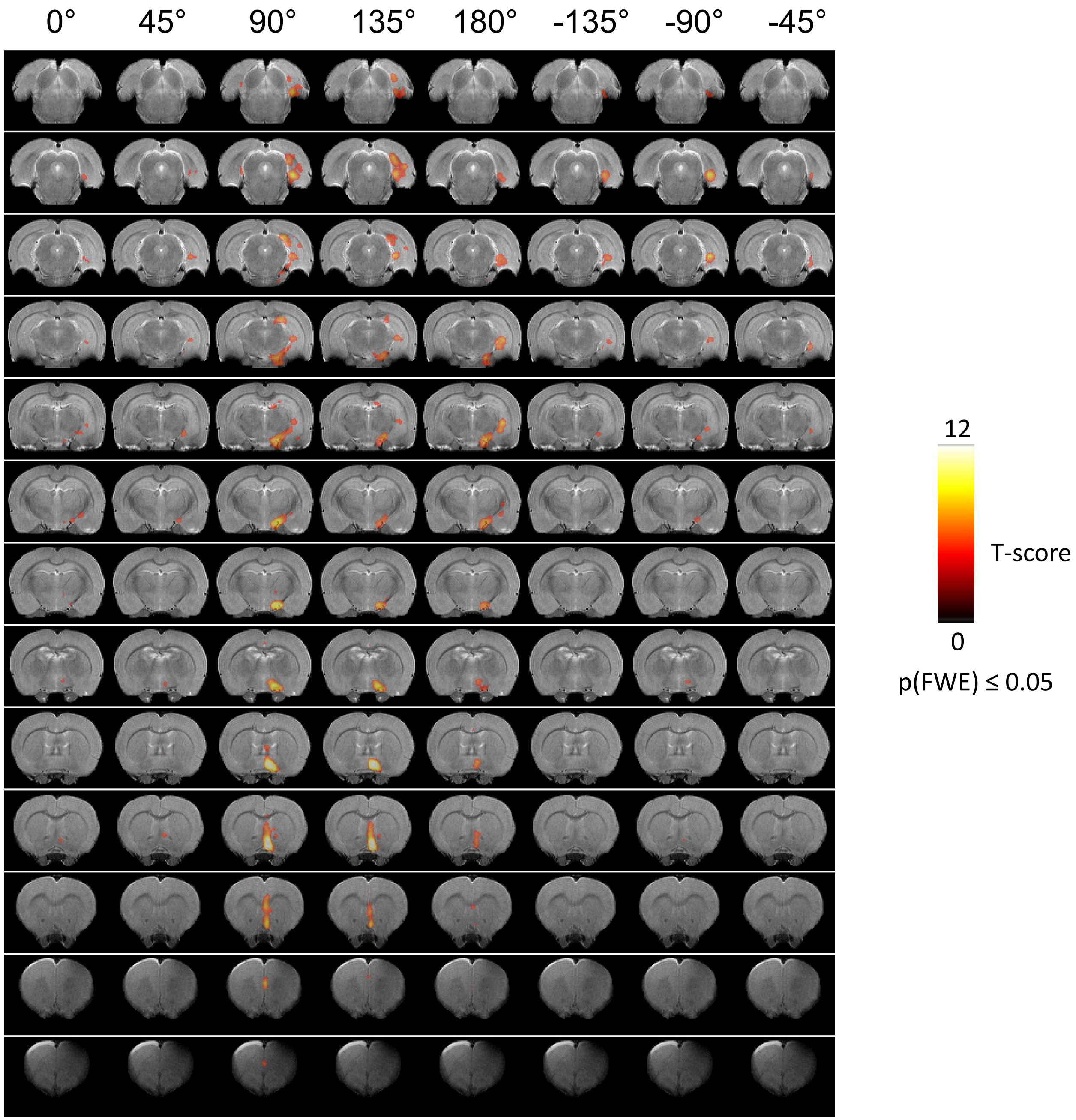

**Order**	**1**	**2**	**3**	**4**	**5**	**6**	**7**	**8**
Angle(°)	180	135	0	90	45	-45	-90	-135

MSN group
Rat 13, 0.7 mA

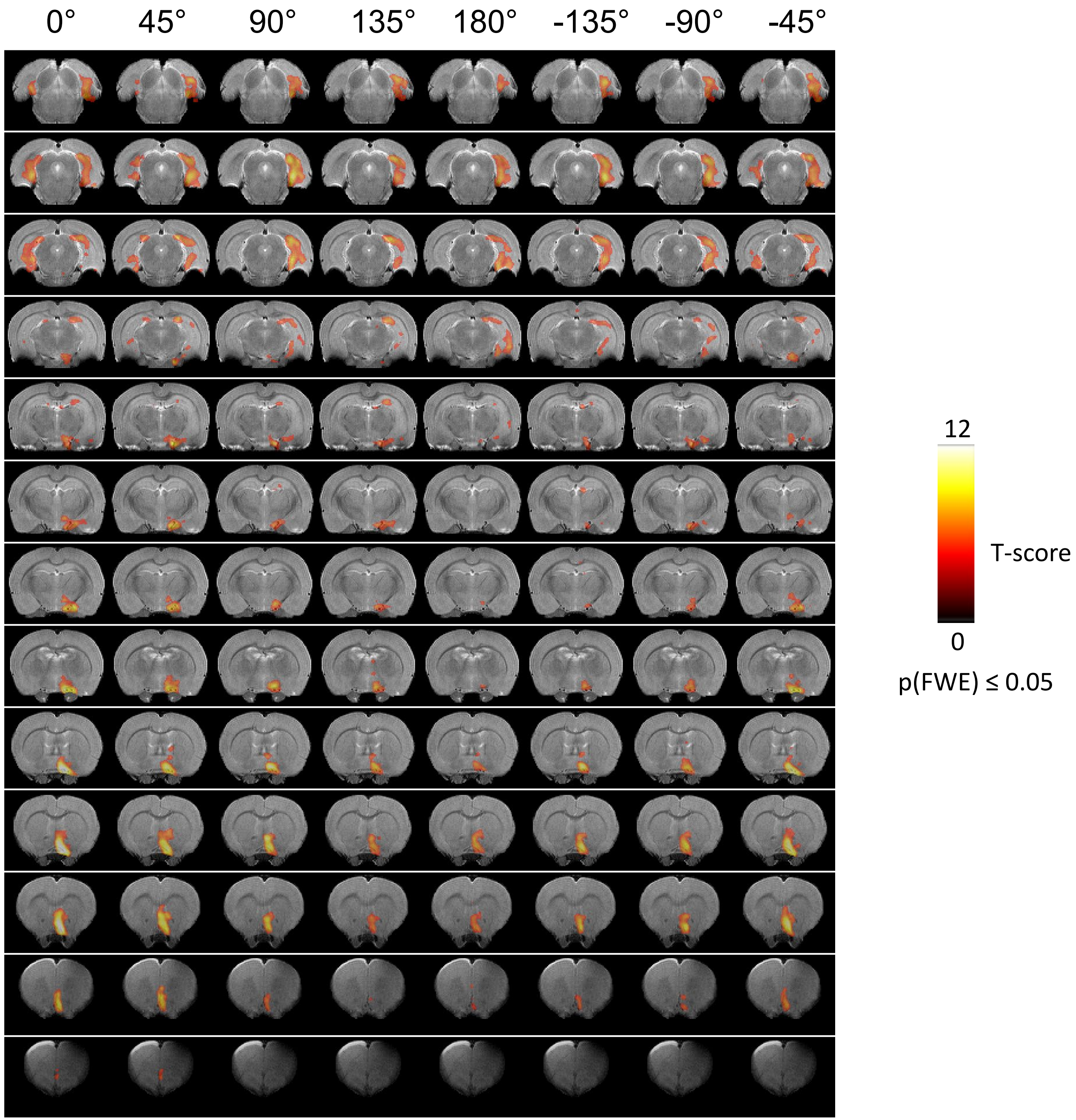

Order	1	2	3	4	5	6	7	8
Angle(°)	180	-45	45	-135	-90	0	90	135

MSN group

Rat 14, 0.8 mA

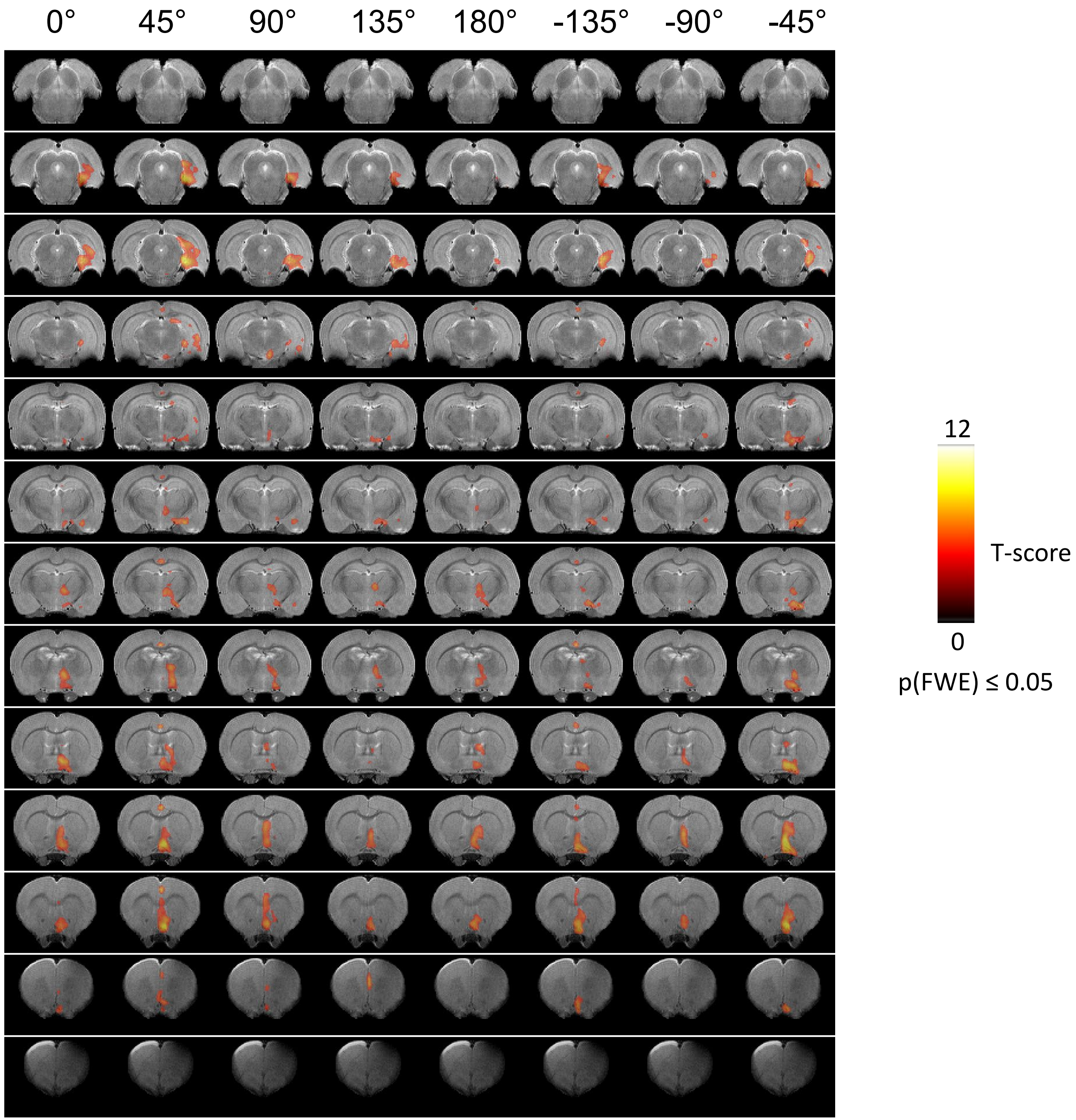

**Order**	**1**	**2**	**3**	**4**	**5**	**6**	**7**	**8**
Angle(°)	135	90	-135	-45	180	0	-90	45

MSN group
Rat 17, 1.3 mA

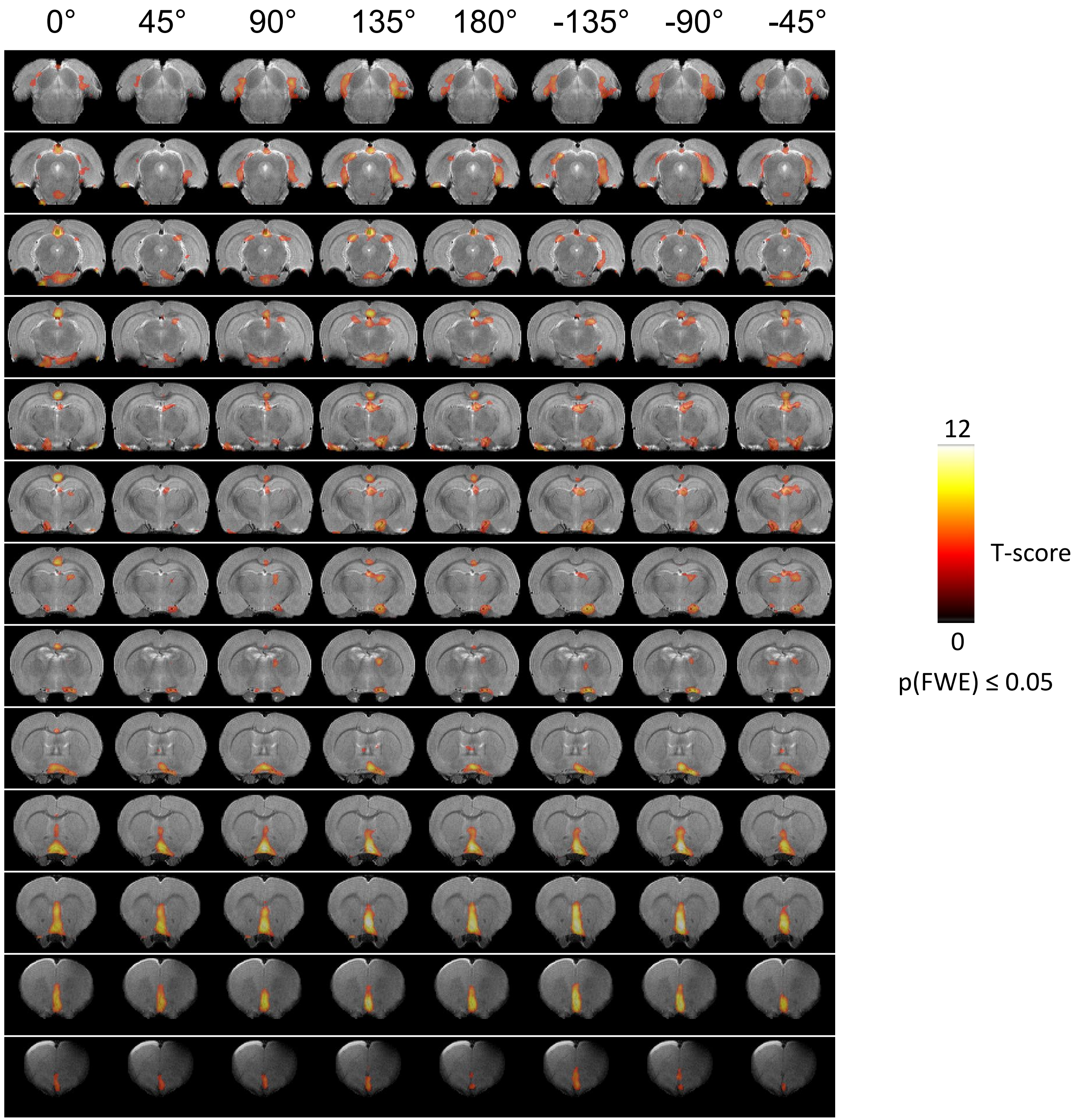

**Order**	**1**	**2**	**3**	**4**	**5**	**6**	**7**	**8**
Angle(°)	135	90	180	-90	0	-45	-135	45

MSN group

Rat 16, 1.15 mA

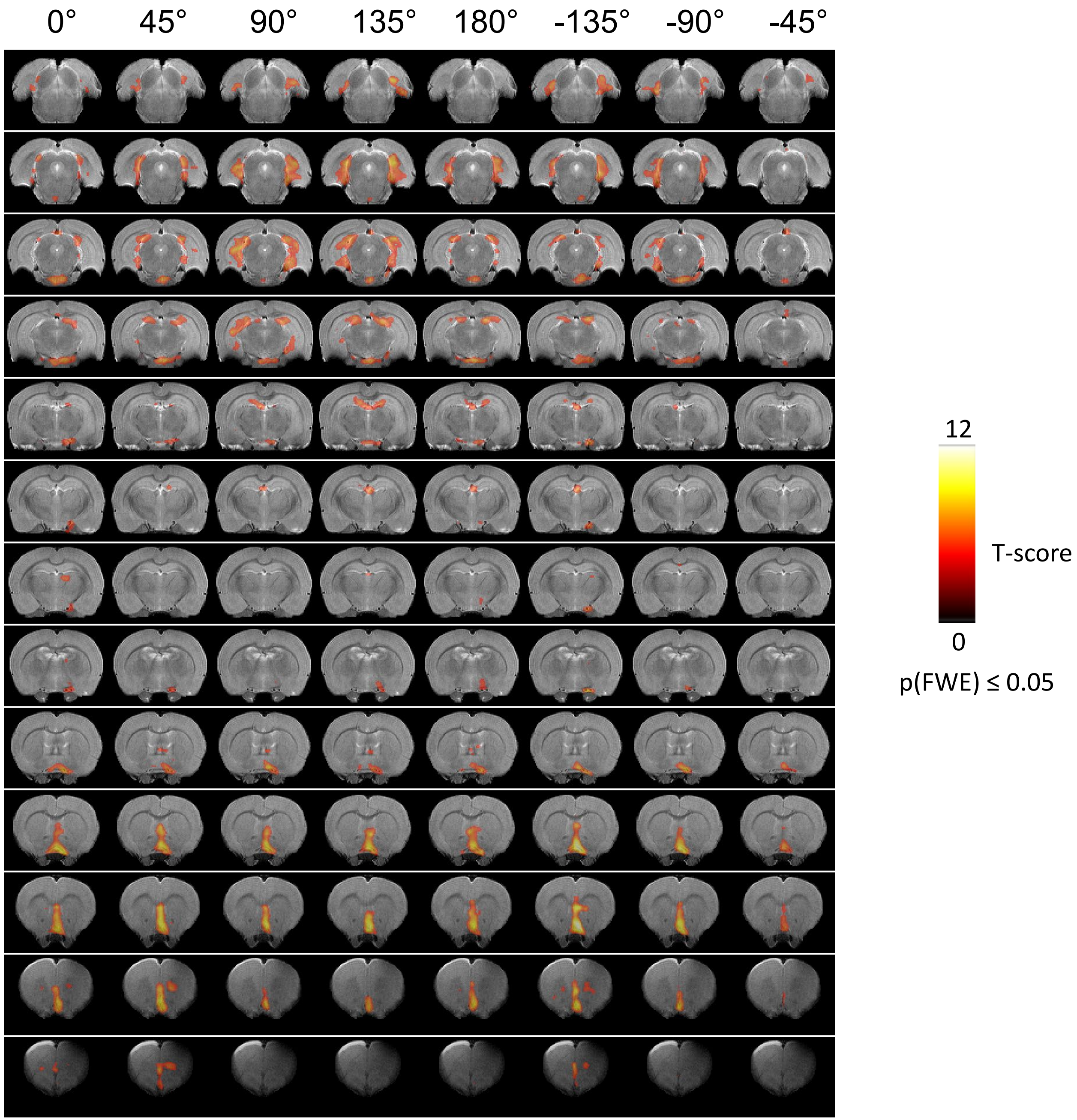

**Order**	**1**	**2**	**3**	**4**	**5**	**6**	**7**	**8**
Angle(°)	-90	-45	-135	90	0	180	135	45

MSN group
Rat 17, 1.0 mA

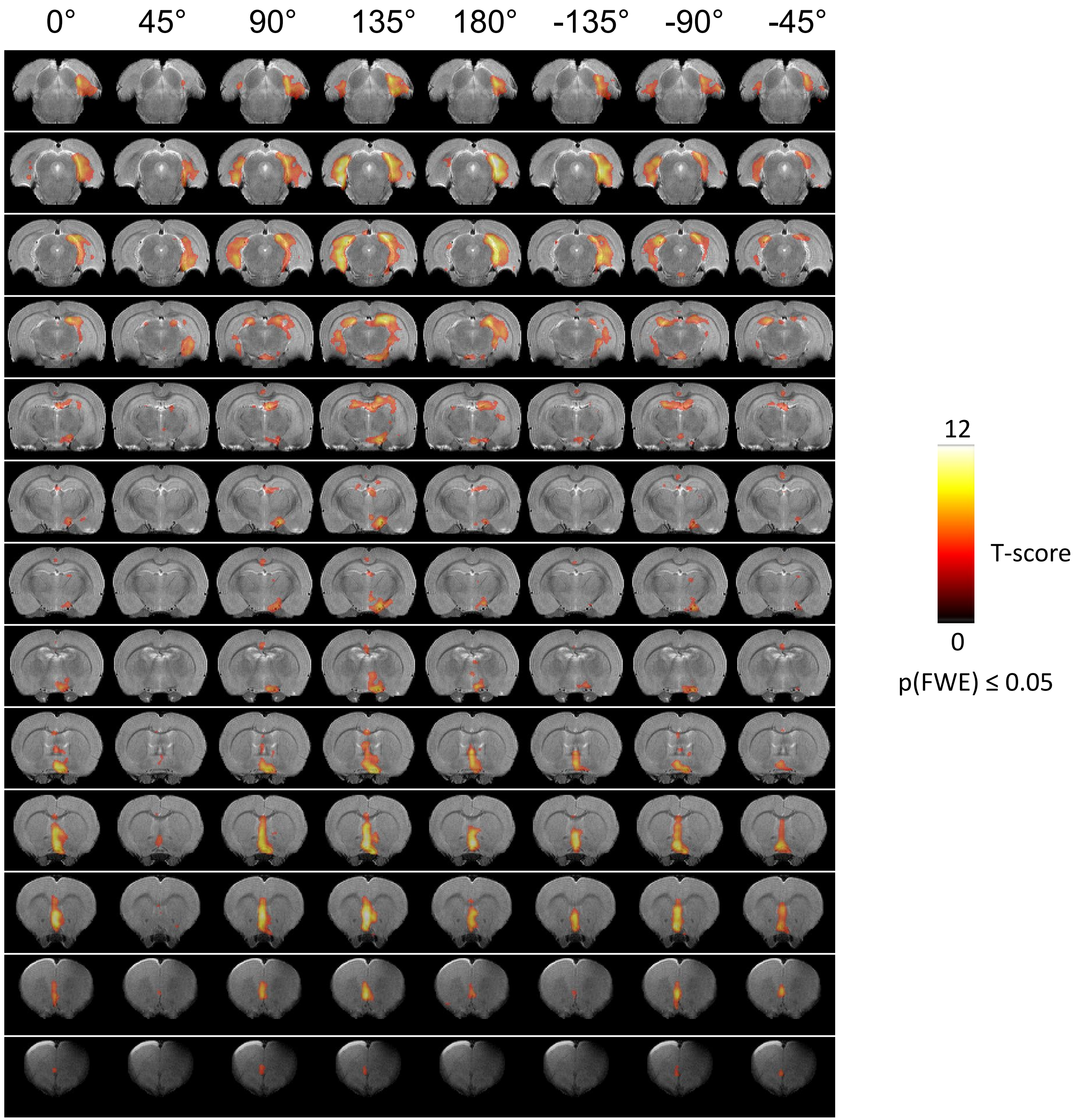

Order	1	2	3	4	5	6	7	8
Angle(°)	-135	0	45	90	180	-45	135	-90

MSN group

Rat 18, 1.25 mA

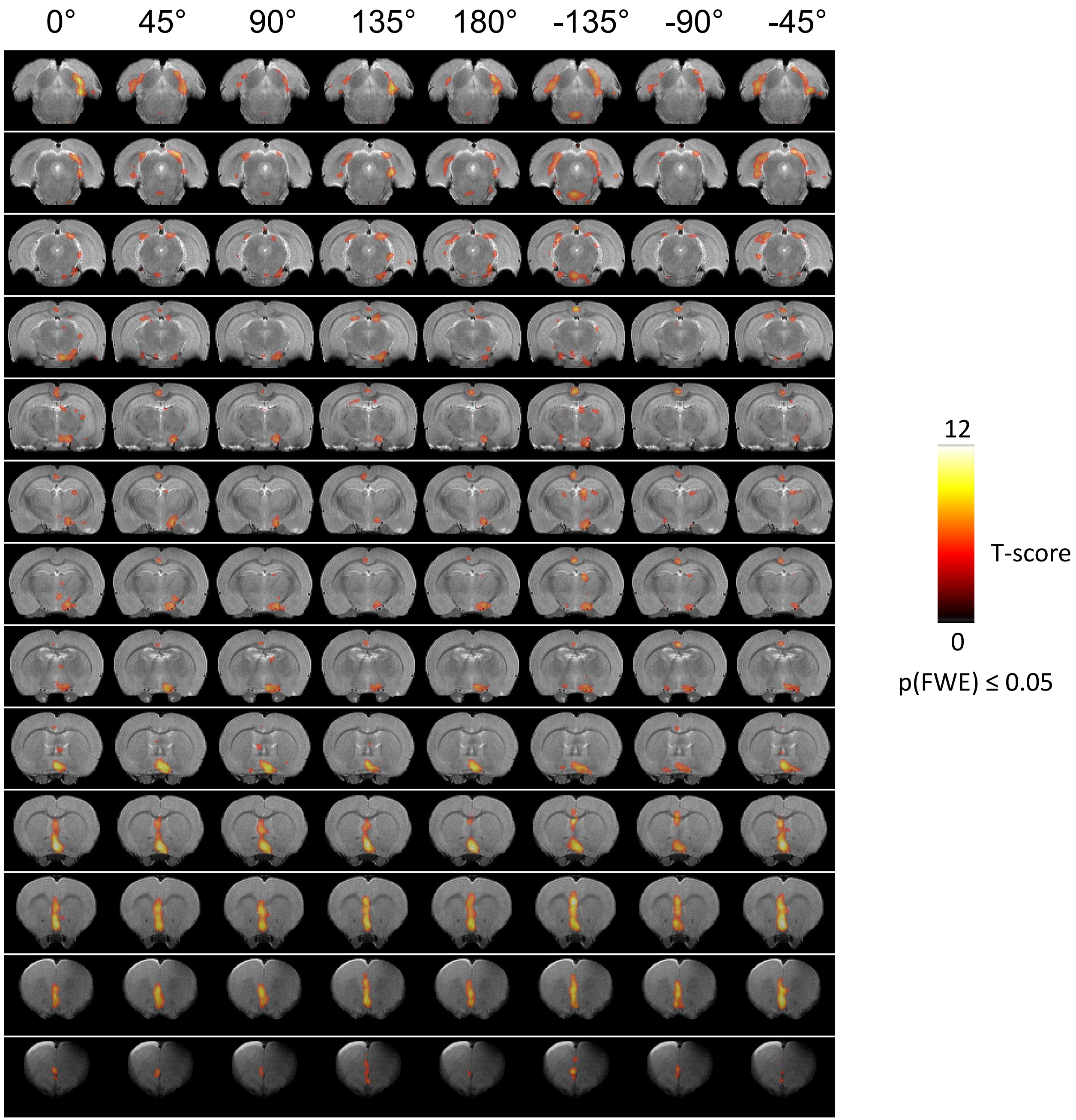

**Order**	**1**	**2**	**3**	**4**	**5**	**6**	**7**	**8**
Angle(°)	-90	135	0	-45	45	90	180	-135
